# Epididymal extracellular vesicles harbor and convey mRNA to sperm for transfer to zygotes

**DOI:** 10.1093/nar/gkag330

**Published:** 2026-04-20

**Authors:** Natalie A Trigg, Grace S Lee, Alexis G Leach, Colin C Conine

**Affiliations:** Division of Neonatology, Children’s Hospital of Philadelphia, Philadelphia, PA 19104, United States; Departments of Genetics and Pediatrics—Penn Epigenetics Institute, Institute of Regenerative Medicine, and Center for Women’s Health and Reproductive Medicine, University of Pennsylvania Perelman School of Medicine, Philadelphia, PA 19104, United States; Centre for Reproductive Biology, School of Science, The University of Newcastle, Awabakal Country, Callaghan, NSW 2308, Australia; Division of Neonatology, Children’s Hospital of Philadelphia, Philadelphia, PA 19104, United States; Departments of Genetics and Pediatrics—Penn Epigenetics Institute, Institute of Regenerative Medicine, and Center for Women’s Health and Reproductive Medicine, University of Pennsylvania Perelman School of Medicine, Philadelphia, PA 19104, United States; Pharmacology Graduate Group, University of Pennsylvania Perelman School of Medicine, Philadelphia, PA 19104, United States; Division of Neonatology, Children’s Hospital of Philadelphia, Philadelphia, PA 19104, United States; Departments of Genetics and Pediatrics—Penn Epigenetics Institute, Institute of Regenerative Medicine, and Center for Women’s Health and Reproductive Medicine, University of Pennsylvania Perelman School of Medicine, Philadelphia, PA 19104, United States; Cellular and Molecular Biology Graduate Group—Genetics and Epigenetics, University of Pennsylvania Perelman School of Medicine, Philadelphia, PA 19104, United States; Division of Neonatology, Children’s Hospital of Philadelphia, Philadelphia, PA 19104, United States; Departments of Genetics and Pediatrics—Penn Epigenetics Institute, Institute of Regenerative Medicine, and Center for Women’s Health and Reproductive Medicine, University of Pennsylvania Perelman School of Medicine, Philadelphia, PA 19104, United States

## Abstract

The epididymis plays a critical role in promoting sperm maturation, including remodeling the sperm RNA payload. While small RNAs have been extensively studied in this context, the epididymal contribution to larger sperm RNAs, such as messenger RNAs (mRNAs), remains underexplored. This is largely due to the translational quiescence of mature spermatozoa and the hypothesis that these RNAs are residual by-products of spermatogenesis. Yet, mRNAs carried by sperm have been detected in the zygote, indicating they may act beyond fertilization. However, whether epididymal somatic cells contribute mRNAs to sperm, as they do small RNAs, has not been experimentally examined. Here, we provide a comprehensive analysis of the mRNA landscape of mouse sperm, epithelial cells, and extracellular vesicles (EVs) isolated from the proximal (caput) and distal (cauda) epididymis. Through this analysis and sperm-EV co-incubation experiments, we demonstrate the transfer of mRNAs from epididymal EVs to sperm. Further, through sperm RNA microinjection into zygotes, we uncover gene regulation in the early embryo driven by the introduction of sperm RNAs, specific to >200-nucleotide RNA species. These findings reveal the dynamic mRNA profile of sperm that is delivered to the egg and demonstrate that RNA species beyond small RNAs are capable of influencing preimplantation embryo gene expression.

## Graphical Abstract

**Graphical Abstract:**
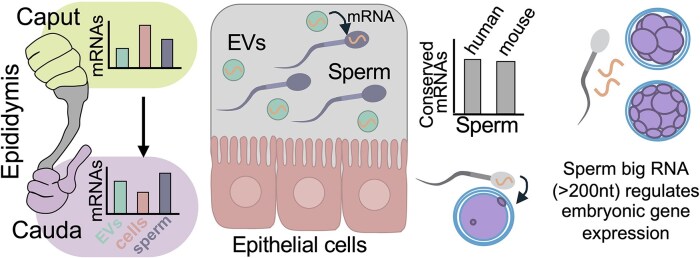

## Introduction

Mature spermatozoa harbor a complex array of RNAs, including small non-coding RNAs [microRNA (miRNA), piRNAs, and transfer RNA (tRNA)-derived RNAs (tDRs or also known as tRFs)] and long non-coding RNAs (lncRNAs), as well as coding messenger RNAs (mRNAs) [1–4]. During germ cell development, the RNAs present in sperm reflect the transcription required for sperm development. However, at the culmination of spermatogenesis, particularly the differentiation of haploid round spermatids to differentiated spermatozoa (spermiogenesis), genomic compaction and the loss of key organelles precludes *de novo* RNA synthesis [5, 6]. Thus, all RNAs present in sperm must be transcribed during spermatogenesis, processed from spermatogenic precursor transcripts, or acquired non-autonomously. It is widely thought that the majority of sperm mRNAs arise from transcription during spermatogenesis and remain in the differentiated spermatozoa. Indeed, highly abundant mRNAs detected in mature epididymal sperm are also detected at high abundance in germ cells during spermatogenesis in the testis (i.e. *Prm1* and *2*) [7–9]. Numerous studies have detected a rich profile of mRNA transcripts within the sperm of multiple species, including humans and mice [1, 10]. However, whether these RNAs are, as thought, simply the remnants of the spermatogenic gene expression program or if they have functions post-fertilization in the zygote is not thoroughly understood. Post-fertilization functions of sperm RNAs have been proposed. For example, sperm *Igf2* mRNA expression correlates with embryo morphokinetics in humans, and this relationship was recently probed mechanistically by microinjecting synthesized *Igf2* mRNA into mouse parthenotes and assessing gene expression [11]. Microinjection of *Igf2* mRNA into mouse parthenotes resulted in altered expression of 99 genes in the early embryo. Similarly, other sperm-delivered mRNAs, such as *Dby* have been implicated in zygotic development through microinjection of antisense RNA to *Dby* mRNA, which prohibits embryonic development to the two-cell stage in mice [12]. Moreover, sperm RNAs >200 nucleotides have been shown to be modulated by paternal stress exposure and contribute to specific non-genetically inherited trauma phenotypes in offspring, including food intake and glucose response to insulin [13]. Such findings highlight a functional role for sperm-delivered mRNAs in the embryo and uncover another layer of non-genetic information harbored by spermatozoa.

The accumulation of mRNAs in mature spermatozoa outside of spermatogenic persistence, such as through post-transcriptional processing or non-autonomous accumulation in the epididymis, has yet to be thoroughly addressed. While certain types of sperm RNAs could be processed from spermatogenic precursors in the epididymis, such as small RNAs, the de novo generation of mRNAs in epididymal sperm is unlikely as they are typically processed co-transcriptionally [14]. However, the acquisition of mRNAs during epididymal transit from the epididymal epithelium is plausible, as this mechanism has been clearly demonstrated to occur for small RNAs [15–17]. The epididymis is a long, coiled tubule situated on the posterior surface of the testis and facilitates sperm maturation following sperm development in the testis. During transit through the epididymis, sperm acquire motility and undergo membrane remodeling and other maturational changes that render them capable of fertilization [18]. Beyond these functional gains, spermatozoa also acquire macromolecules during epididymal transit. A notable example is the increased abundance of 57 sperm proteins, including carbonic anhydrase 4 and calcium-binding tyrosine phosphorylation-regulated protein, as well as post-translational modifications to existing proteins [16, 17, 19–21]. Additionally, it has been shown that the small RNA profile of sperm is dramatically remodeled as sperm passage from the proximal (caput) epididymis to the distal (cauda) epididymis [16, 22]. Specifically, a subset of miRNAs are transferred to sperm during the transition from the caput to cauda epididymis and, importantly, these miRNAs were demonstrated to regulate preimplantation embryonic gene expression [15, 23, 24]. Interestingly, miRNAs and tDRs are thought to be delivered to maturing sperm by first being packaged into extracellular vesicles (EVs), called epididymosomes, within the epididymal epithelium [16, 17, 25]. However, alternative mechanisms could also function to transfer RNAs from the epididymis to sperm, including direct delivery in association with RNA-binding proteins, through nanotubules [26], or not directly from the epididymal epithelium but via RNA shuttling through the cytoplasmic droplet [27]. It should be noted that none of the mechanisms are mutually exclusive and could function simultaneously to hone the RNA profile of sperm prior to conception.

Although the mRNA profile of epididymal sperm has not previously been comprehensively assessed across the epididymis, evidence suggests that, like other macromolecules, it may change during epididymal transit, with transcripts potentially gained and lost between the caput and cauda epididymis [28]. This challenges the notion that sperm mRNAs are merely remnants of testicular development and suggests that a mechanism exists for epididymal sperm to acquire and lose mRNAs. If packaged with mRNAs, the EVs produced by the epididymal epithelium could provide a mechanism of mRNA acquisition by epididymal sperm. Moreover, the presence of big RNAs (>200 nucleotides), likely to include mRNAs, lncRNAs, and circular RNAs (circRNAs), has been observed in epididymal EVs using gel electrophoresis [23]. These findings led us to hypothesize that EVs deliver mRNA to sperm during epididymal transit. To assess this, we investigated the mRNA profile of EVs, epithelial cells, and sperm isolated from the caput and cauda epididymis via mRNA-seq. In extending this analysis to understand the contribution of EVs to the sperm mRNA profile, we also performed co-incubation experiments to demonstrate that EVs transfer mRNA to sperm *in vitro*. Lastly, to begin uncovering the functional role of sperm RNAs longer than 200 nucleotides, we used parthenogenetically activated eggs combined with microinjection to evaluate how different fractions of sperm RNA influence embryonic gene expression.

## Materials and methods

### Animals

Adult FVB/NJ male mice (8–12 weeks old) and female mice (5–7 weeks old) were derived from mice purchased from Jackson Labs (Strain #:001800) and breed in-house. All animals used in this study were handled and kept in conditions under strict compliance with the Children’s Hospital of Philadelphia and the University of Pennsylvania Institutional Animal Care and Use Committee regulations (CHOP IACUC Protocol #23-001364, UPenn PSOM IACUC Protocol #806911).

### Sperm and extracellular vesicle isolation

Testicular sperm was obtained from mouse testis tissue suspensions using isotonic Percoll solution as previously described [15]. Epididymides were dissected from male mice and separated into the proximal caput epididymis (including the initial segment) and the distal cauda epididymis (Supplementary Fig. S1B). Dissected epididymal tissue was placed in a dish containing 1.5 ml of TYH media [120 mM NaCl, 4.7 mM KCl, 1.7 mM CaCl_2_2H_2_O, 1.2 mM KH_2_PO_4_, 1.2 mM MgSO_4_, 25 mM NaHCO_3_, 5.5 mM glucose, 0.5 mM sodium pyruvate, 10 µg/ml gentamicin, 3.0 mg/ml bovine serum albumin (BSA), and 1.0% phenol red]. The luminal contents of the cauda epididymis were gently squeezed into the media under a dissection microscope, being careful to minimize tissue disruption. For the caput epididymis, small incisions were made into the tissue to release the luminal fluid into the media. Tissue was removed from the dish and sperm was allowed to disperse for 10 min at 37°C. For cauda epididymal sperm isolation, after incubation, the media was transferred to a microcentrifuge tube and sperm were allowed to swim up for 10 min at 37°C, before filtering over a 70 µm filter and transferred to a new tube and centrifuged 5 min at 500 × *g* for 5 min. Following this centrifugation, the supernatant was transferred to a new tube and subjected to differential centrifugation to isolate EVs as previously described [15]. Sperm pellets were retained to purify populations of sperm. Cauda sperm pellets were washed in phosphate-buffered saline (PBS), incubated in somatic cell lysis buffer on ice for 10 min, and subsequently washed prior to snap-freezing. Sperm washes following somatic cell lysis was performed at 10 000 × *g* at 4°C to pellet all cells and minimize cell loss. Caput epididymal sperm pellets were resuspended in 1 ml of media following removal of supernatant and layered over a 28% Percoll gradient and centrifuged for 15 min, 400 × *g* at 37°C. Pelleted sperm were washed in PBS and any remaining somatic cells were removed by incubation in somatic cell lysis buffer for 10 min on ice. Sperm were washed a final time in PBS and snap-frozen. Incubation in somatic cell lysis buffer (0.01% sodium dodecyl sulfate, 0.05% Triton X-100) also removed adherent cytoplasmic droplets from spermatozoa, which ensured we assayed the population of RNAs in mature sperm (Supplementary Fig. S1D and E).

EV isolation was confirmed using nanoparticle tracking and negative-stain electron microscopy (Supplementary Fig. S2A and B). Nanoparticle analysis was performed using the Particle Metric ZetaView and negative-stain electron microscopy was conducted by the University of Pennsylvania Electron Microscopy Resource Lab (RRID:SCR_022375).

### RNase treatment of extracellular vesicles

EVs were resuspended in 300 μl of cold PBS and divided equally among treatments. Samples were then incubated in RNase A (0.5 µg/ml in PBS) for 20 min at 37°C to eliminate any unprotected RNA. RNase A activity was inactivated via incubation at −80°C for 5 min, and then vesicle preparations were immediately homogenized in TRI-reagent and total RNA was extracted. Mock treatments were incubated as above with the addition of vehicle only (PBS) at equal volumes in place of RNase A.

### Sperm RNA extraction and preparation for microinjection

RNA was extracted from populations of mature spermatozoa collected from 20 mice as previously described [15]. Total RNA was quantitated, and a portion of total RNA was diluted and aliquoted for injection. The remaining RNA was subjected to size selection (>200 nucleotides) using the Monarch Total RNA Miniprep kit (NEB, T2010S). After size selection of the RNA populations, RNA was diluted to desired concentration, and H3.3-GFP mRNA was added, ready for microinjection.

### Epididymal epithelial cell purification

Epithelial cells were purified from caput and cauda epididymal tissue as previously described [29–31]. Populations of epithelial cells were checked for purity by staining with nuclear stain, DAPI (4′,6-diamidino-2-phenylindole) and recording the percentage of somatic cell purity. Only samples with >80% purity were kept for RNA-seq.

### Sperm-extracellular vesicle co-incubation

Cauda epididymal EVs were isolated from three male mice as outlined above and resuspended in 50 µl of TYH media, mixed well to resuspend vesicles into solution, and kept at room temperature. Spermatozoa were isolated from the caput epididymis of four male mice as described earlier with slight modification. Following incubation after tissue dispersion, the sperm-containing supernatant was immediately filtered over a 70 µm cell strainer and then loaded onto the Percoll gradient. Following centrifugation, sperm was resuspended in TYH media and washed twice before resuspension in 200 µl media and split into two. EVs (50 µl) were added to one tube and 50 µl of media to the control and allowed to incubate for 2 h at 37°C with constant slow rocking. At the completion of the co-incubation, 850 µl of PBS was added and sperm were pelleted for 5 min at 500 × *g*. Cells were incubated in somatic cell lysis buffer for 7 min and washed in PBS before snap-freezing as previously described [4]. Centrifugation following somatic cell lysis was performed at 10 000 × *g* for 5 min at 4°C.

### *In vitro* fertilization and zygote collection

Female 5–7-week-old FVB/NJ mice were superovulated by intraperitoneal injection of 5 IU pregnant mare serum gonadotropin and 5 IU of human chorionic gonadotropin (hCG) 48 h later. Cumulus-oocyte complexes (COCs) were retrieved from the distal oviductal ampullae 13–15 h after hCG injection and recovered in warmed PBS. COCs were then divided into two, where the first was added to a droplet of human tubal fluid (HTF) supplemented with 1.0 mM reduced glutathione (GSH) ready for *in vitro* fertilization (IVF), while the remaining COCs were dissociated via brief incubation in hyaluronidase (3 mg/ml), and eggs washed of any residual cumulus cells and incubated in KSOM (Merck, Cat# MR-101-D) for 30 min under an atmosphere of 5% O_2_, 5% CO_2_. After equilibration, cumulus cell-free eggs were collected into lysis buffer [1 × TCL buffer (Qiagen, Cat# 1070498) supplemented with 1% β-mercaptoethanol] and frozen in preparation for RNA-seq. Spermatozoa were collected from the cauda epididymis as described earlier and allowed to swim out in Biggers, Whitten, and Whittingham media (BWW; composed of 91.5 mM NaCl, 4.6 mM KCl, 1.7 mM CaCl_2_2H_2_O, 1.2 mM KH_2_PO_4_, 1.2 mM MgSO_4_7H_2_O, 25 mM NaHCO_3_, 5.6 mM D-glucose, 0.27 mM sodium pyruvate, 44 mM sodium lactate, 5 U/ml penicillin, 5 μg/ml streptomycin, 20 mM HEPES buffer, and 3.0 mg/ml BSA containing 1.0 mg/ml of methyl-β-cyclodextrin) for 45 min at 37°C under an atmosphere of 5% O_2_, 5% CO_2_. Following capacitation, spermatozoa were added to the COC-containing droplet and incubated for 3 h. After co-incubation, presumptive zygotes were thoroughly washed free of any bound spermatozoa and cultured in KSOM. To collect zygotes prior to the initiation of zygotic genome activation, one-cell embryos were collected 4.5 h after fertilization, a time point when zygotic genome activation has yet to commence [32, 33]. Only zygotes with two pronuclei (fertilized) were collected into lysis buffer and stored at −80°C for RNA-seq.

### Parthenogenesis, microinjection, and embryo culture

COCs from 12 superovulated female mice (per replicate) were collected as described earlier into a single dish of filtered PBS. COCs with an estimated egg count of ∼20 (typically 1–2 COCs) were immediately transferred to a drop of supplemented HTF for IVF as described earlier. The remaining COCs were incubated in hyaluronidase to remove cumulus cells (see earlier). Following removal of cumulus cells, eggs were washed in KSOM before transfer to a droplet of KSOM supplemented with 10 mM SrCl_2_, 4 mM EGTA (ethyleneglycol- bis(β-aminoethyl)-N,N,Nʹ,Nʹ-tetraacetic acid), and cytochalasin B at a final concentration of 0.05 µg/ml to activate by incubation at 37°C under an atmosphere of 5% O_2_, 5% CO_2_ for 1 h [34]. Following activation, parthenotes were washed through six droplets of KSOM ready for microinjection.

Parthenotes were transferred to the injection plate, in droplets of flushing and holding media supplemented with 1% polyvinyl alcohol for micromanipulation. Control injections consisted of H3.3-GFP mRNA alone (50 ng/ul), and experimental injections included total RNA (100 ng/ul) or big RNA (50 ng/ul) isolated from populations of mature mouse spermatozoa. All experimental RNA injections also had H3.3-GFP mRNA added to injection mix at a final concentration of 50 ng/ul. RNA injections were carried out using a Femtojet (Eppendorf/Calibre) microinjector with Femtotip II microinject capillary tips at 100 hPa pressure for 0.2 s, with 7 hPa compensation pressure [35]. Under these microinjection settings, this corresponds to delivery of ~53 fL of volume per embryo, equating to 5.3 fg of total RNA and 2.65 fg of big RNA, respectively. After RNA injection, parthenotes were washed in KSOM and cultured. The presence of GFP signal was confirmed using fluorescence microscopy, and any negative two-cell embryos were removed from the culture dish. Injected embryos were cultured and collected for single-embryo RNA-sequencing. Four-cell and morula-stage embryos were collected into lysis buffer at 46 and 72 h post-fertilization, respectively.

### RNA-sequencing and analysis

RNA was extracted from mouse sperm, epithelial cells, and EVs as previously described [15, 16, 36]. Contaminating DNA was removed from total RNA samples by incubation with DNase I (Qiagen, 79254) as per the manufacturer’s instructions. RNA was quantified using Qubit fluorometer, and 2 ng of RNA was used for RNA-seq library generation. For embryos, following lysis, RNA was isolated via RNAClean-XP beads (Beckman Coulter, Cat# A63987) as outlined previously [37]. Libraries were generated for sequencing using the SMART-seq protocol as previously described [15, 38]. Data were mapped to the *Mus musculus* reference genome (mm10) using Feature counts on ViaFoundry [39] and normalized to transcripts per million (TPM). Sequencing quality control was assessed by determining the number of transcripts within each sample with TPM ≥ 1. Sperm, epithelial cell, and EV samples with <5500 and egg/embryo samples with <8000 genes satisfying this cutoff were removed. Raw read counts were uploaded to R statistical software, and using the DESeq2 package, data were normalized and differential expression of genes was determined using false discovery rate *P*-value [40]. All datasets were further filtered to obtain a list of “expressed/detected” genes within each sample. This filtering involved removing any transcripts with <5 TPM average in the groups being compared. Except for embryo datasets, further filtering involved the removal of transcripts with less than half of replicates exhibiting TPM ≤ 2 (Supplementary Tables S1–S12 and S15–S17). Eulerr package in R was used to determine the overlapping genes among different groups and generate Venn diagrams. Aligned RNA-seq reads (BAM files) were analyzed using RSeQC package and bedtools coverage tool to compute coverage for metagene coverage plots and annotated gene regions, respectively.

### Interactive web application development

**Figure 1.**
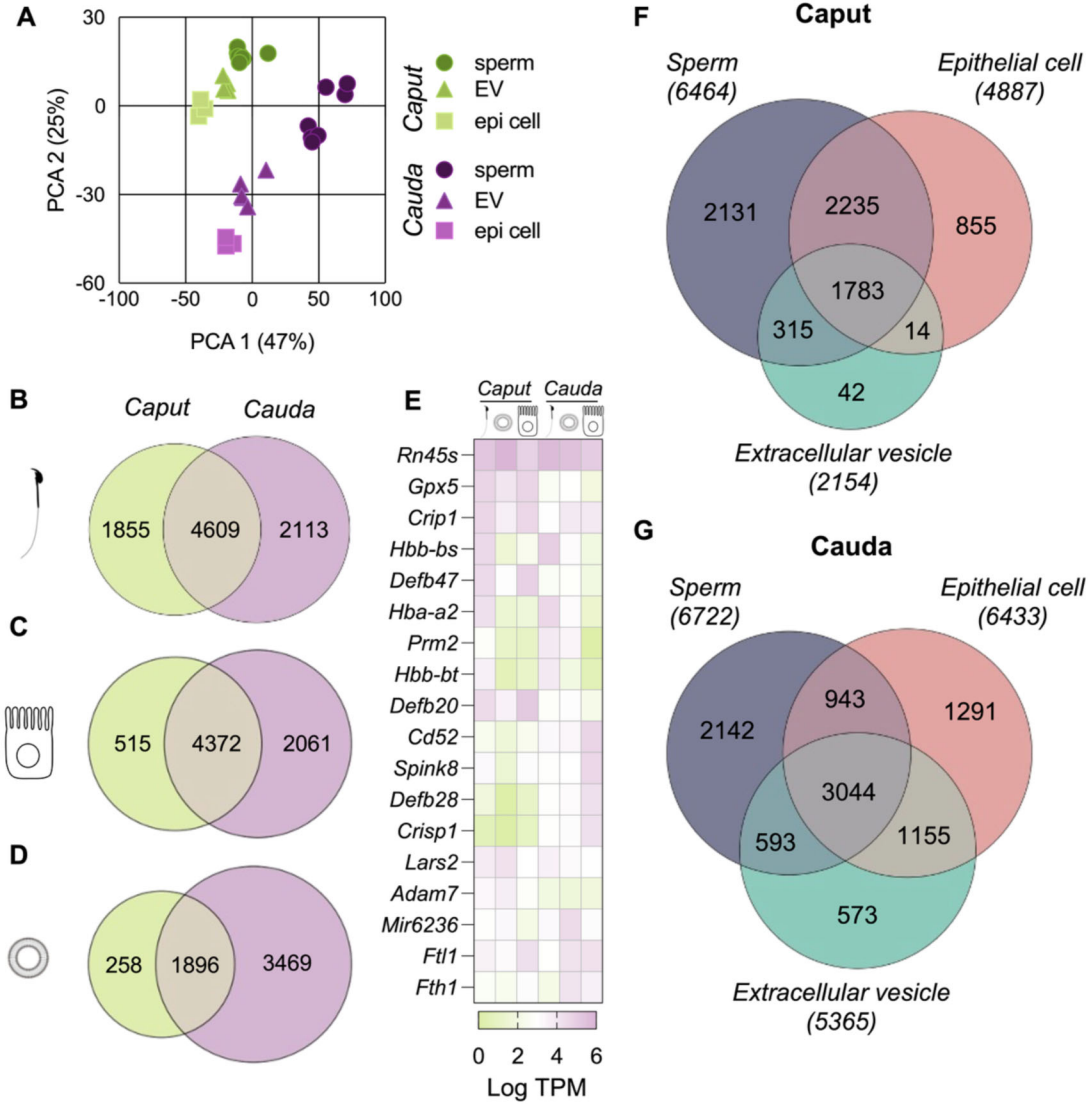
Segment-specific expression of epididymal mRNAs in sperm, epithelial cells, and epididymosomes. (**A**) Principal component analysis (PCA) of the transcriptome of 38 individual samples of sperm, epithelial cells (epi cell), and epididymal EVs isolated from the caput (green) and cauda (purple) epididymis. A minimum of three biological replicates were sequenced (*n* = 3–8), with each biological replicate containing pooled samples from 3 to 4 mice. Proportional Venn diagram comparing the mRNA profile of genes expressed in panel (**B**) sperm, (**C**) epithelial cells, and (**D**) EVs isolated from the caput and cauda epididymis. (**E**) Heatmap depicting the expression (log TPM) of the top five most abundant genes across sample type and epididymal segment. Proportional Venn diagrams illustrating the number of identified genes expressed in populations of sperm, epithelial cells, and EVs isolated from (**F**) the caput and (**G**) the cauda epididymis. Raw gene lists for each sample were filtered to determine the list of “detected” genes, which were compared here. Genes with TPM ≤ 2 in ≥50% of replicates were removed, and the list was further filtered to remove genes with average TPM value of ≤5 across all replicates.

**Figure 2.**
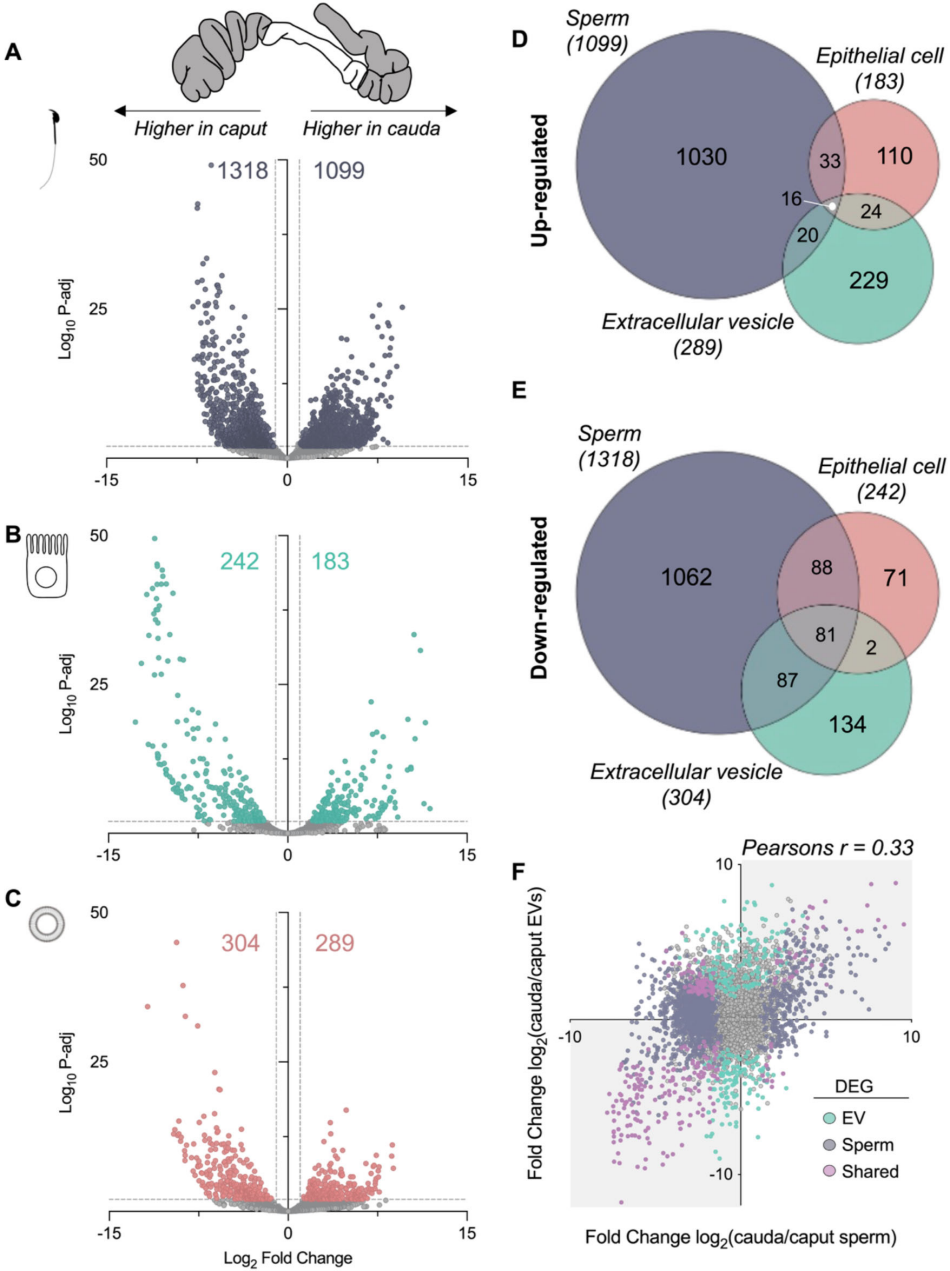
The mRNA profile of sperm, epithelial cells, and epididymal extacellular vesicles (EVs) is modulated along the epididymis. Volcano plots depicting log_2_ fold change and log_10_ adjusted *P*-value of identified genes in populations of (**A**) sperm, (**B**) epithelial cells, and (**C**) EVs isolated from the caput and cauda epididymis. Colored dots indicate differentially abundant genes as determined using DESeq2 in R. Significance threshold: fold change ± >2 and adjusted *P*-value of ≤ .01. Venn diagrams illustrating the overlap of (**D**) up-regulated and (**E**) down-regulated genes in sperm, epithelial cells, and EVs when cauda samples were compared to caput samples. (**F**) Correlation plot of cauda/caput log_2_ fold change of mRNA expression in sperm (*x*-axis) and EVs (*y*-axis). Colored dots highlight DEGs in sperm (purple), EVs (green), and those genes altered in both samples (pink) when cauda samples are compared to caput.

Alongside the inclusion of our processed RNA-seq data as supplementary files, we have developed an interactive web-based resource (Shiny App ) that allows real-time exploration of our epididymal RNA-seq data presented in Figs 1 and 2. This tool builds on previous platforms such as ShinySperm [41] and ShinyPlacenta [42] and was created using the shiny package (v 1.12.1) in R Studio (v 2025.09.1) with base R (v 4.5.2). Data handling and visualization are supported by DT, ggplot2, ggiraph, ggVennDiagram, dplyr, tidyr, and shinythemes. The complete code supporting this application, named “Epididymal mRNA Explorer” is available on GitHub (https://github.com/N-A-Trigg/EpiRNA).

### Assessment of RNA conservation in human sperm

Human sperm low-input mRNA-seq data were used to determine the conservation of sperm mRNAs between mice and humans. Utilizing the Ensembl (release #13) Bioconductor BiomaRt package for R, a list of all human orthologs to mouse genes was created. Gene common names used during sequence mapping were converted to Ensembl IDs using BiomaRt (external gene name, external synonym, and NCBI RefSeq ID were searched). During this conversion, 218 gene names could not be matched to an Ensembl ID using BiomaRt and were therefore excluded, and genes returning multiple Ensembl IDs for the same gene name were removed. Human orthologs were then called for each gene in the total list of genes mapped during sequencing. Abundance of human orthologs was calculated and plotted against mouse gene abundance for the mRNAs detected in mature mouse sperm.

## Results

### The mRNA profile of epididymal sperm, epithelial cells, and extracellular vesicles is modified along the epididymis

To quantify the mRNA expression dynamics that occur in epididymal sperm, we performed mRNA-seq on caput (proximal) and cauda (distal) epididymal sperm, epididymal epithelial cells, and EVs isolated from epididymal fluid from mice. Importantly, these experiments confirm the presence of mRNAs within epididymal sperm total RNA preparations (Supplementary Fig. S1A). While small RNA delivery from EVs to sperm during epididymal transit is well established [16, 17, 23], the role of EVs in transferring big RNAs (>200 nucleotides), particularly mRNAs, remains unexplored. Further, we have previously shown abundant big RNA species are present in EVs isolated from the caput and cauda epididymis via denaturing polyacrylamide gel electrophoresis of total RNA [23]. Thus, we hypothesize that epididymal EVs harbor mRNAs with dynamic accumulation along the epididymis that could deliver mRNAs to sperm.

PCA grouped biological replicates together and separated samples by sample type and epididymal segment (Fig. 1A). Determination of Pearson correlations highlighted similar mRNA expression in sperm and EVs, while epithelial cells were distinct (Supplementary Fig. S1C). Further, the mRNA profile of sperm, epithelial cells, and EVs isolated from the caput epididymis were distinct from those isolated from the cauda epididymis. In exploring the dynamic changes that occur in mRNA profiles along the epididymis, we compared filtered gene lists (see “Materials and methods” section) of sperm, epithelial cells, and EVs from the caput and cauda epididymis. This comparison revealed that over 50% of transcripts in sperm and epithelial cells are present in both segments of the epididymis (Fig. 1B and C). Compared to sperm and epithelial cells, epididymal EVs harbored significantly fewer mRNA species (Fig. 1D). Moreover, in EVs, only 33.7% of transcripts (1896 transcripts of total 5623 identified in EVs) were shared between the caput and cauda epididymis. Rather, significantly more transcripts were detected in EVs isolated from the cauda epididymis [61.7% (3 469 transcripts) of mRNAs were unique to cauda] compared to the caput epididymis [4.6% (258 transcripts) unique to caput; Fig. 1D]. Examination of the five most abundant genes across sample type and epididymal segment highlighted segment-specific genes (e.g. *Defb20* and *Crisp1*) and sample type-specific genes (e.g. *Prm2* and *Flt1*; Fig. 1E). Next, we compared detected transcripts across sample types from the same segment to determine shared and unique genes (Fig. 1F and G). For both epididymal segments examined, sperm harbored the largest number of unique transcripts that were not detectable in either epithelial cells or EVs (>2000 mRNAs unique to sperm). The percentage of transcripts expressed in all three sample types was comparable between the caput (24.2%) and cauda (31.2%) epididymis. Moreover, EVs displayed the least number of unique transcripts of all sample types, with the majority of EV transcripts (98.0% in the caput and 89.3% in the cauda epididymis) also identified in other samples (Fig. 1F and G). Importantly, to provide a visual tool to explore these results, we developed an interactive web application (Epididymal mRNA Explorer ) that allows users to query and visualize the datasets underlying these analyses.

Despite being transcriptionally silent, mature spermatozoa isolated from the distal cauda epididymis harbor many unique transcripts. Hence, we next sought to establish the dynamic changes in the RNA profile along the epididymis. Using DESeq2, we determined differentially expressed/abundant genes (DEGs) within sperm, epithelial cells, and epididymosomes isolated from the caput and the cauda epididymis. This analysis revealed the differential accumulation of 2417 genes in caput epididymal sperm compared to cauda epididymal sperm (Fig. 2A). Of these genes, 1318 were more abundant in caput epididymal sperm, while the remaining 1099 were detected at higher levels in cauda epididymal sperm (Fig. 2A). The expression profile of epithelial cells and EVs was also significantly altered along the epididymis. Epithelial cells isolated from the cauda epididymis displayed altered expression of 425 genes: 183 of which exhibited increased expression, while 242 decreased in expression compared to caput epithelial cells (Fig. 2B). In line with this, mRNA from EVs also differed depending on the epididymal location, with mRNAs from 289 genes significantly increased in cauda epididymal EVs compared to caput, while 304 were decreased (Fig. 2C). Comparison of DEGs across sample types revealed that while a portion of mRNAs were equally altered in all sample types from the caput to the cauda epididymis, the majority of DEGs were unique to each sample type (Fig. 2D and E; Supplementary Fig. S1G–I). Despite this, correlating the caput-to-cauda epididymis fold change (log_2_) of mRNAs in sperm and EVs revealed a significant correlation (Pearson’s correlation coefficient of 0.33, Fig. 2F). These findings establish that the epididymal abundance dynamics of many mRNAs in sperm are similar between sperm and EVs transiting the epididymis. To improve data reliability, a stringent filtering step (see “Materials and methods” section) was applied to minimize false positive detection of mRNAs, a common challenge in low-input transcriptomics. While this approach improves confidence in the results, it is important to note as a remaining limitation to the data. To further increase confidence in the transcripts identified and generate a high-confidence list of transcripts harbored by sperm originating from the epididymis, we independently sequenced testicular sperm populations to compare to mature cauda epididymal sperm populations. This approach identified 2500 transcripts detected in cauda epididymal sperm but not in testicular sperm (Supplementary Fig. S1F and Supplementary Table S18). Further, 390 of these transcripts increase in abundance in cauda epididymal sperm compared to caput epididymal sperm (Supplementary Fig. S1F).

### Extracellular vesicles convey mRNAs to sperm following *in vitro* co-incubation

**Figure 3.**
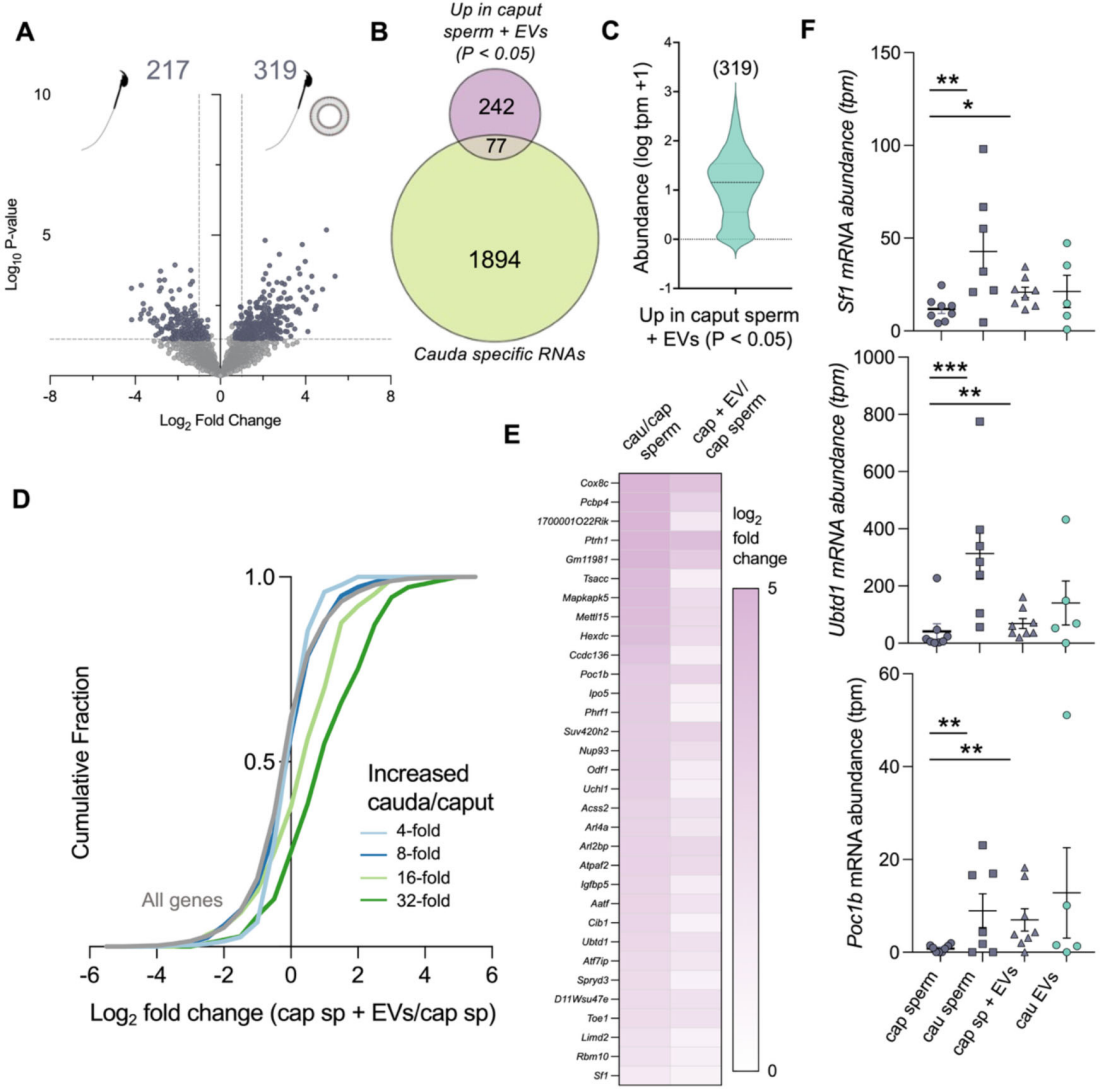
Epididymal EVs convey mRNAs to sperm following *in vitro* co-incubation. (**A**) Volcano plot depicting the RNA changes between caput sperm (mock incubation) and caput sperm co-incubated with cauda EVs *in vitro*. Colored dots indicate differentially expressed genes. Threshold for determining differentially expressed genes was fold-change of ±1.5 and *P*-value ≤ .05. (**B**) Venn diagram depicting cauda-specific genes (not detected in caput sperm and >10 average TPM in cauda sperm) and up-regulated RNAs in caput sperm co-incubated with EVs compared to caput sperm alone. (**C**) Violin plot of mRNA abundance in cauda epididymal EVs of the 319 mRNAs increased in caput epididymal sperm incubated with EVs. (**D**) Cumulative distribution plot (CDF) of log_2_ fold change between caput sperm only and caput sperm incubated with EVs for all genes (gray line) and genes that are significantly increased in cauda sperm compared to caput sperm (green and blue lines). Increased genes are separated into four bins based on cauda sperm/caput sperm fold change data from Fig. 2A. (**E**) Heatmap illustrating the log_2_ fold change of the 32 genes significantly increased, both in cauda sperm and caput sperm incubated with EVs, compared to caput sperm alone. (**F**) mRNA abundance (TPM) of Sf1, Ubtd1, and Poc1b in caput and cauda epididymal sperm, caput sperm post-co-incubation experiment, and cauda EVs. Each dot indicates an individual biological replicate, and statistical analysis was performed to determine differences between caput and cauda sperm and caput sperm with and without co-incubation of EVs. Asterisks indicate level of significance, where *P-value < .05, **P-value < .01, and ***P-value < .001.

Based on our findings that mRNA accumulation in EVs and sperm exhibit strikingly similar dynamics during epididymal transit and, importantly, the previous demonstration of small RNA and protein transfer between sperm and EVs, we performed *in vitro* co-incubation experiments of caput epididymal sperm and cauda epididymal EVs to determine if EVs transfer mRNAs to epididymal sperm [16, 17, 43]. Following co-incubation and thorough washing, RNA was extracted from populations of sperm co-incubated with EVs and naïve sperm with control co-incubation. This analysis revealed the increased abundance of mRNAs from 319 genes in sperm co-incubated with EVs and 217 genes with decreased abundance (Fig. 3A). Intriguingly, comparing the 319 increased genes to the list of cauda epididymal sperm-specific transcripts, mRNAs at ≥5 TPM in cauda but not caput epididymal sperm, we identified 77 genes shared between these groups (Fig. 3B). Moreover, 92% (219 mRNAs) of the 319 mRNAs increased following co-incubation were also detected in cauda EV preparations (Fig. 3C). Owing to the technical challenges in mimicking epididymal sperm exposure to EVs (2 h *in vitro* co-incubation compared to 7–10 days of exposure *in vivo*), to quantitate more subtle alterations in sperm mRNAs, we sorted genes increased in abundance in cauda epididymal sperm compared to caput epididymal sperm into bins grouped by fold enrichment in cauda epididymal sperm compared to caput epididymal sperm. Then, we examined the fold change of these groups between control caput epididymal sperm and caput epididymal sperm incubated with EVs (Fig. 3D). Plotting cumulative distribution reveals that the genes with the greatest increase from caput to cauda epididymal sperm naturally (*in vivo*) demonstrated increased abundance in populations of caput epididymal sperm incubated with EVs compared to caput epididymal sperm mock-incubated controls. Notably, the transcripts of 32 genes that demonstrated a statistically significant increase when caput epididymal sperm were exposed to EVs compared to control incubated caput epididymal sperm reached levels comparable to that observed naturally in cauda epididymal sperm. These mRNAs include *Sf1, Ubtd1*, and *Poc1b*, which are also abundant in cauda epididymal EVs (Fig. 3E and F).

### mRNAs are protected in extracellular vesicles

**Figure 4.**
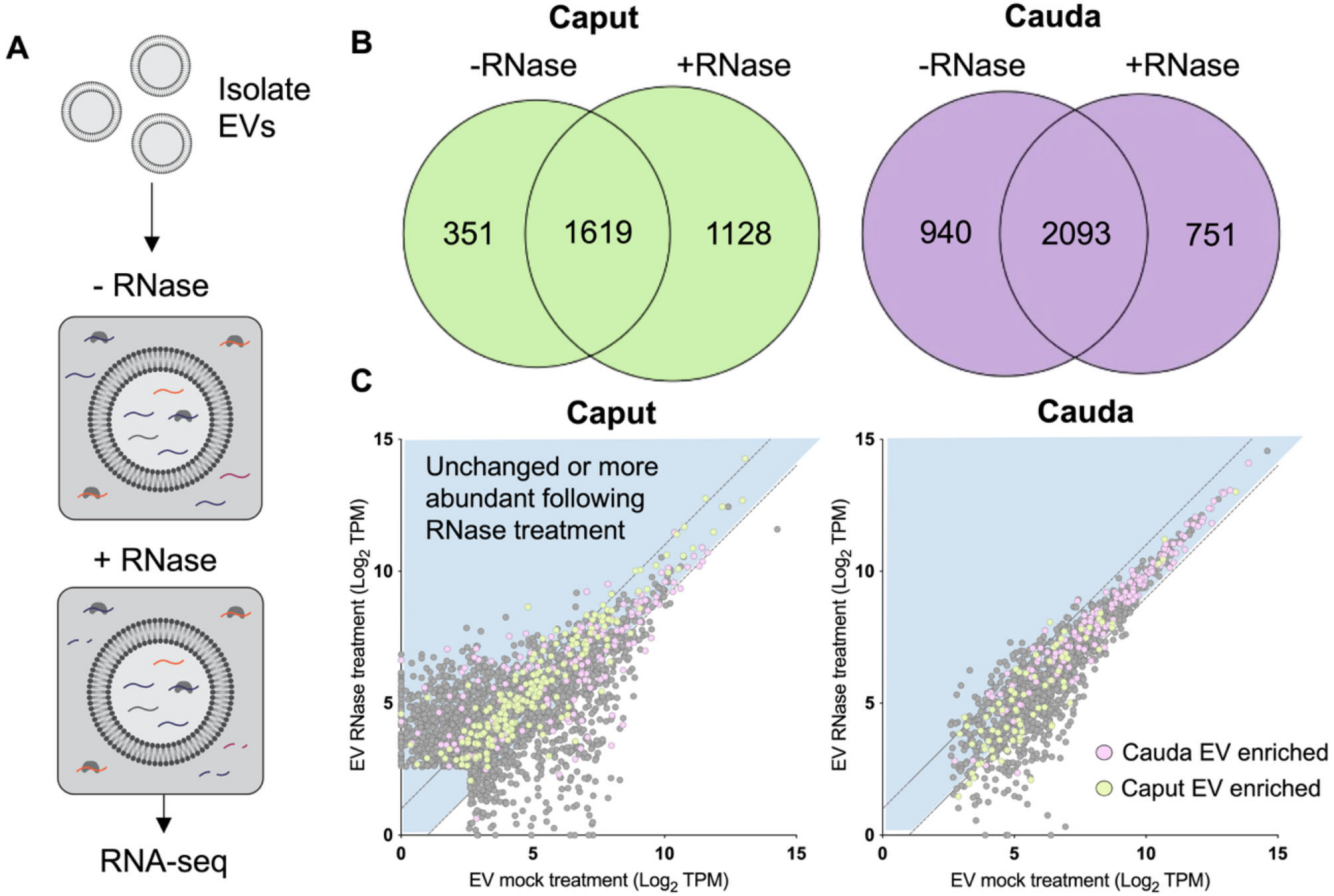
Epididymal mRNAs are protected in EVs. (**A**) Preparations of EVs isolated from the caput and cauda epididymis were split into equal aliquots and one sample was treated with RNase A, and the other mock-incubated without the addition of RNase. RNA was extracted following treatment, and libraries generated for mRNA-seq. (**B**) Venn diagrams illustrating the overlap of detected transcripts between mock and RNase treated preparations. (**C**) Scatter plots of average log_2_ TPM for mock EV treatments (*x*-axis) versus RNase-treated EVs (*y*-axis). Colored dots highlight mRNAs shown to be significantly altered in EVs between the caput and cauda epididymis. Purple indicates mRNAs enriched in cauda EVs, and green indicates mRNAs enriched in caput EVs. The genes that lie in the blue shaded portion of the scatter plot highlight those that are either unchanged or enriched in EV preparations following RNase treatment.

The enrichment of EVs using the protocol in this study does not preclude the possibility of mRNA captured arising from unprotected extracellular mRNA that co-elutes with EVs. Hence, to discriminate EV-encapsulated RNA from extracellular RNA not associated with EVs, we treated EVs with RNase A to remove any free RNA, using previously published methods (Fig. 4A; [44]). Comparison of the detected RNAs in populations of EVs incubated in the presence of vehicle (mock) or RNases revealed the majority of mRNAs in both caput and cauda epididymal EVs were detected in both mock and RNase treated samples (Fig. 4B). Examining the average abundance of RNAs within each of these samples showed an enrichment of mRNAs following treatment in populations of caput epididymal EVs, while cauda epididymal EV preparations treated with RNases demonstrated similar abundance to untreated, both consistent with RNase resistance or protection (Fig. 4C). While not impacting vesicle integrity or morphology (Supplementary Fig. S2B), RNase treatment did lead to the depletion of transcripts, suggesting their presence outside of the EVs. Together, these results demonstrate that the mRNA sequenced in our EV preparations is derived from RNA encapsulated within EVs. Importantly, this includes mRNAs we showed to be differentially regulated within EVs from the caput epididymis compared to the cauda epididymis (colored dots, Fig. 4C).

### Sperm transcripts not detected in the egg are found in the zygote

**Figure 5.**
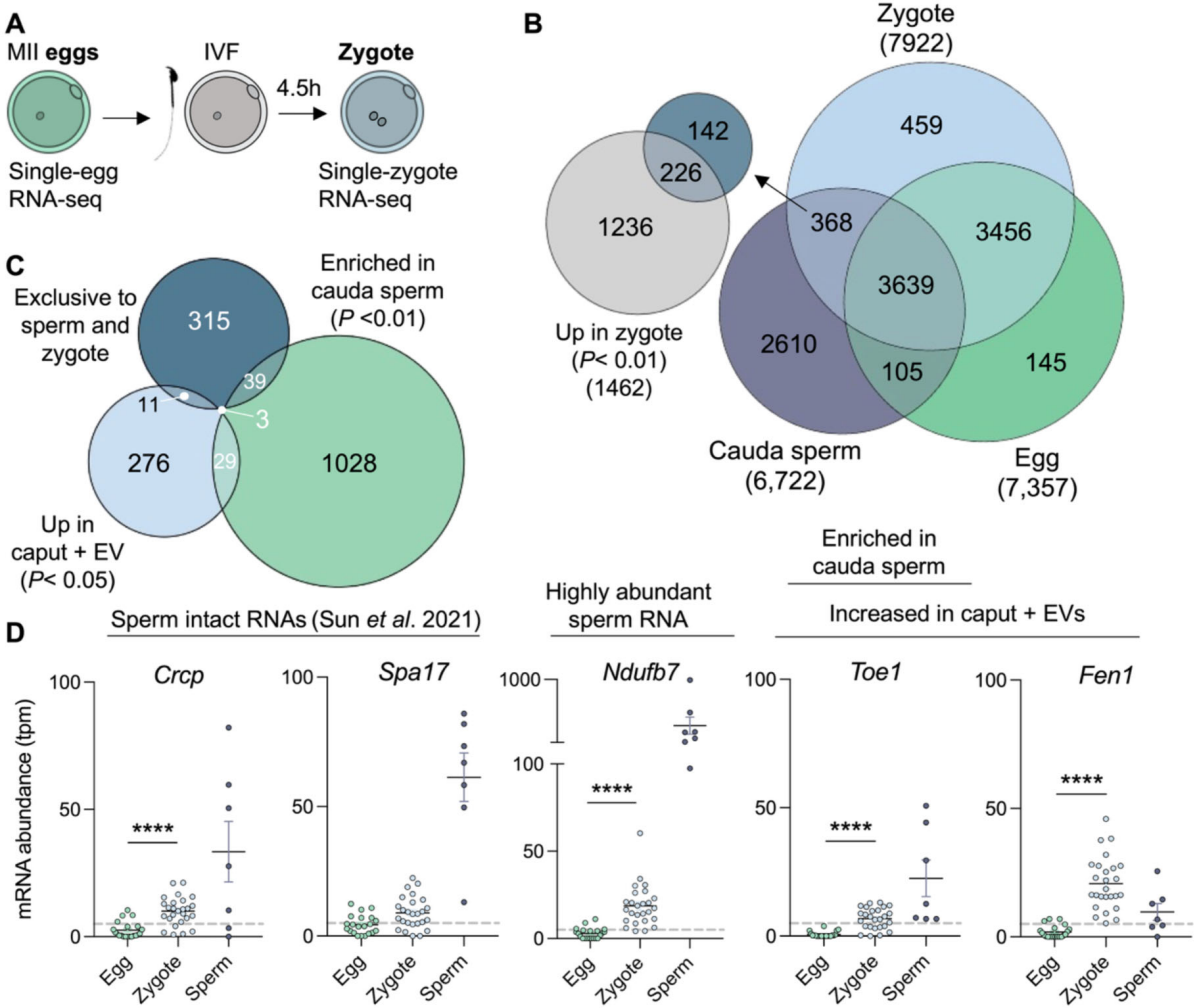
RNAs harbored by sperm are detected in fertilized eggs. (**A**) Experimental schematic for collection of MII mouse eggs and fertilized eggs (zygotes). (**B**) Venn diagram illustrating the overlap of detected genes in mature sperm, MII eggs, and zygotes. Each gene list was filtered to generate a list of expressed genes (see “Materials and methods” section). Inset smaller Venn diagram illustrates the number of shared genes between those exclusively detected in sperm and zygotes and genes significantly increased in the zygote compared to the egg (*P* < .01). (**C**) Overlap of genes exclusively detected in sperm and zygotes, genes enriched in cauda sperm compared to caput sperm, and those identified to be significantly increased in abundance in caput sperm following co-incubation with cauda EVs. (**D**) Abundance of individual RNA transcripts in egg, zygote, and sperm datasets. Individual genes were selected based on the presence in additional gene lists, including sperm intact RNA list [10], highly abundant cauda sperm mRNAs (from Fig. 2A), and increased abundance in caput sperm co-incubated with EVs (from Fig. 3A). Each dot represents a data point from a single egg, zygote or sperm replicate. Horizontal bar represents the mean, and error bars depict the standard error of the mean. Dotted line represents expression threshold of 5 TPM.

In alignment with published literature, we confirmed that sperm carry mRNAs; however, whether these transcripts are delivered to the egg and function following fertilization remains unclear. To investigate this, we compared gene expression profiles of MII eggs and one-cell embryos (zygotes 4.5 h post-fertilization; Fig. 5A and Supplementary Fig. S3A). Using Venn diagrams to compare RNA profiles (following filtering as outlined in Methods) from sperm, eggs, and zygotes revealed most genes were shared among the three groups. Further, the egg and zygote shared the second highest number of expressed mRNAs from specific genes (Fig. 5B). Notwithstanding this, each cell type had a subset of unique mRNAs, with sperm displaying the greatest number of unique mRNAs. Of note, we identified 368 genes that were detected in both sperm and zygotes but not detected in the egg (<5 TPM average). Among these were *Git1, Crcp, Cabyr*, and *Ndufb7*. Further differential expression analysis revealed that 61.4% (226 genes) of these candidates were more abundant, with statistical significance in zygotes compared to eggs (Fig. 5B). Moreover, 42 of these genes were enriched in cauda epididymal sperm compared to caput epididymal sperm, and 14 genes were also transferred to caput epididymal sperm by EVs *in vitro* (Fig. 3A and Supplementary Table S4). Notably, 3 genes (*Toe1, D11Wsu47*e, and *Hexdc*) overlapped across the three of these categories (Fig. 5C), highlighting mRNAs that may be transferred from the soma to sperm during epididymal transit and then to the zygote during fertilization (Fig. 5C).

mRNAs in sperm have been long thought to be remnant degradation products of spermatogenic and spermiogenic gene expression. While our mRNA-seq approach cannot rule out that the mRNAs we identify in sperm are mRNA fragments, this is unlikely because our approach uses polyA-dependent cloning; thus, only polyadenylated RNAs will be sequenced. While gene body coverage plots reveal a 3′ bias in cauda epididymal sperm samples compared to epithelial cells, signal spanning across the gene indicates the presence of transcripts that map along the full-length mRNA (Supplementary Fig. S4A). Moreover, RNA-seq reads from cauda epididymal sperm samples demonstrate coverage across the entirety of specific mRNAs (Supplementary Fig. S4B), including mRNAs exclusively expressed in sperm and zygote (*Ndufb7*). To further address this, we cross-referenced our results with a published dataset of ‘sperm intact RNAs’ (spiRNAs) generated using long-read RNA-seq, confirming 803 spiRNAs in our cauda epididymal sperm dataset (Supplementary Table S13). Of these, 38 were uniquely present in sperm and zygotes but absent from eggs, including *Crcp* and *Spa17*, reinforcing that sperm can deliver intact mRNAs to the egg during fertilization (Fig. 5D).

### Mouse sperm mRNAs are conserved and expressed in human sperm

**Figure 6.**
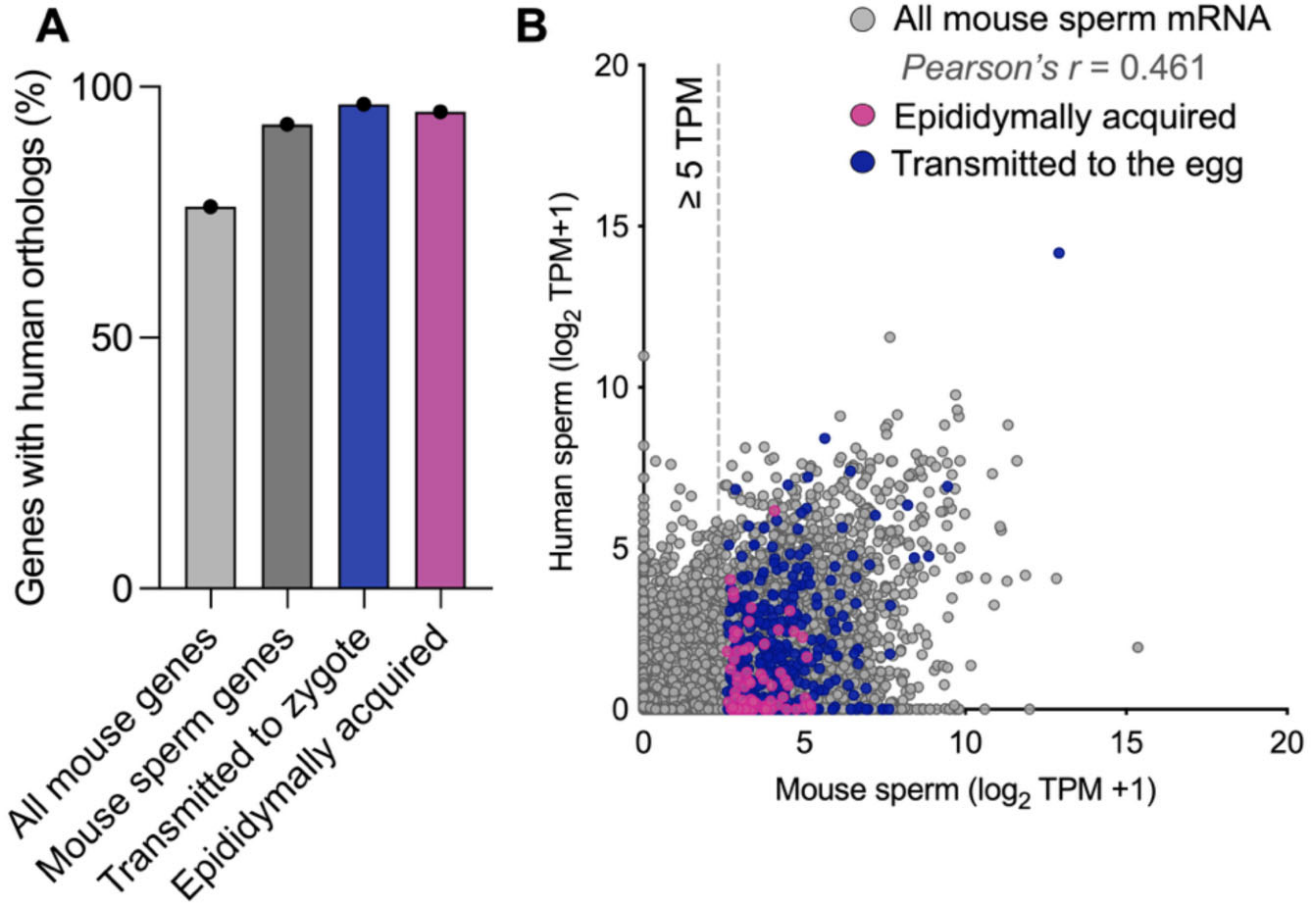
Conservation of mouse sperm mRNAs in human sperm. (**A**) Bar plot illustrating the percentage of mouse sperm mRNAs with orthologs detected in human sperm. Columns depict all mouse genes with human orthologs, all mouse sperm mRNAs, mRNAs determined to be delivered to the egg, and those acquired during epididymal transit. (**B**) Scatter plot illustrating the average mRNA abundance (log_2_ TPM) of the 18 056 mouse sperm mRNAs (unfiltered list) that had human-identified orthologs. Colored dots highlight mRNAs acquired by sperm during epididymal transit (pink) and transmitted to the egg at fertilization (blue).

Following the identification of a population of mRNAs harbored in mouse sperm that are acquired by sperm as they transit the epididymis and delivered to the egg, we sought to examine the conservation of these sperm mRNAs in human sperm. To determine the human orthologs of mouse mRNAs, we used Ensembl’s Bioconductor BiomaRt tool for R. This analysis identified human orthologs for 78.1% of the mouse transcriptome (Supplementary Fig. 6A and Supplementary Table S14). Interestingly, the percentage of mouse mRNAs with human orthologs increased when this analysis focused on mRNAs present in mouse sperm and the subset of mRNAs transmitted to the zygote or epididymally acquired, with 92.5%, 95.3%, and 94.8% conserved, respectively (Fig. 6A). When comparing the abundance of all detected mRNAs in sperm across both species, we observed a significant correlation of abundance (Pearson’s correlation coefficient = 0.132; Fig. 6B), indicating that human and mouse sperm harbor overlapping mRNAs. Moreover, the sperm mRNAs acquired by EV co-incubation and those transmitted to the egg were detected and abundant in human sperm (Fig. 6B). These findings suggest that sperm mRNA epididymal dynamics and the transmission of mRNAs to the zygote during fertilization we observe in mice may be conserved in humans.

### Sperm RNAs regulate postfertilization embryonic gene expression

**Figure 7.**
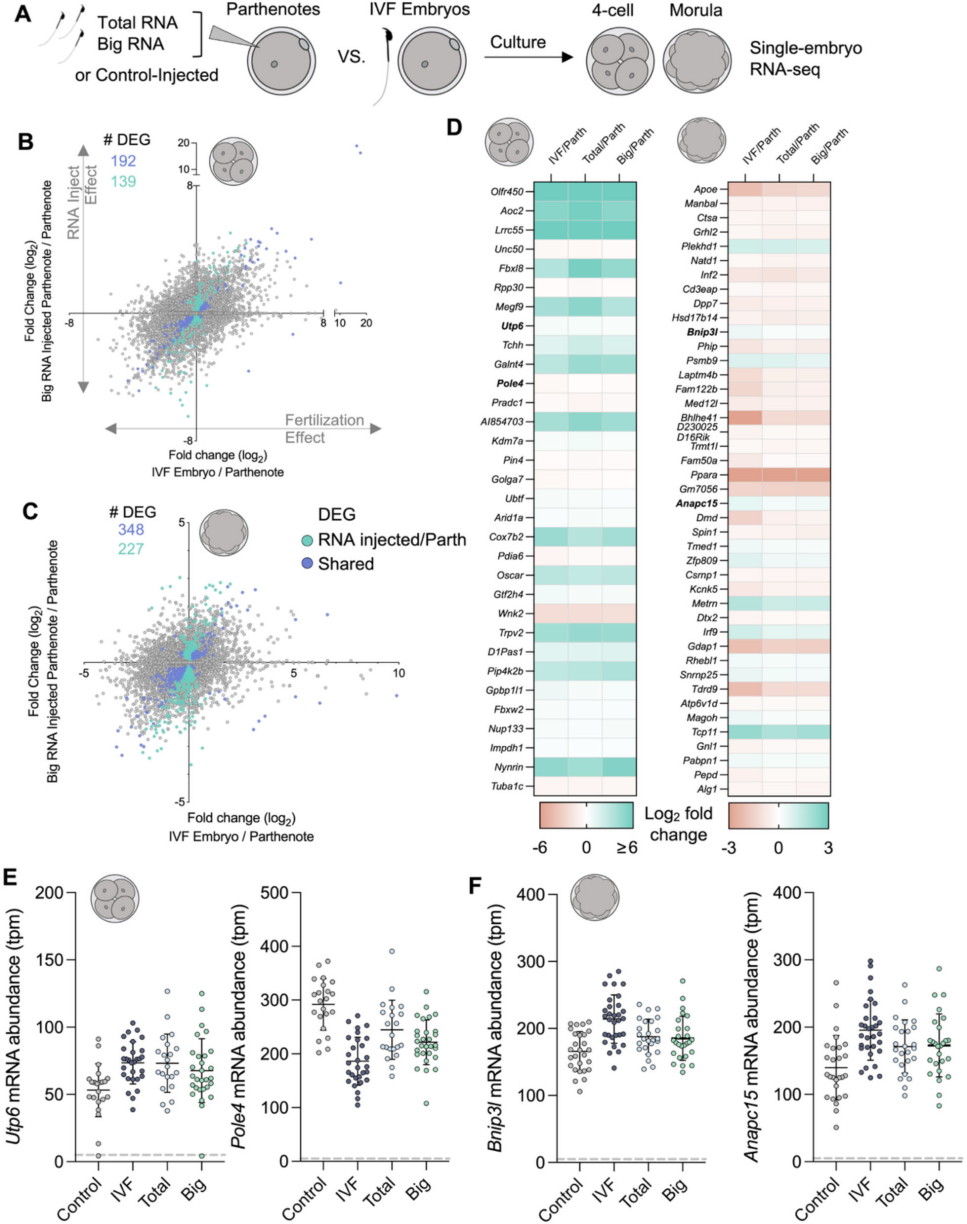
Sperm RNAs influence gene expression in parthenotes. (**A**) Schematic of microinjection experiment involving the injection of sperm RNA into parthenotes and comparison to matched set of IVF generated embryos. (**B, C**) Correlation plots comparing the log_2_ fold change of genes between IVF produced embryos and parthenotes (*x*-axis) to big RNA-injected parthenotes and control parthenotes. Data for (B) four-cell and (C) morula. Colored dots indicate differentially expressed genes (DEGs) for big RNA-injected parthenotes compared to control parthenotes (blue), and those that were similarly altered in IVF embryos compared to control parthenotes (purple). (**D**) Heat map of genes displaying similar expression changes in RNA-injected parthenotes to IVF embryos when compared to control parthenotes. Log_2_ fold change of gene expression in four-cell and morula embryos are depicted. mRNA abundance of individual embryos at (**E**) four-cell and (**F**) morula stage for genes that are altered in RNA-injected parthenotes compared to control parthenotes akin to IVF embryos. Horizontal line represents average TPM and error bars represent standard deviation.

Our data demonstrate the dynamic mRNA profile of sperm along the epididymis, the contribution of EVs to the acquisition of sperm mRNAs, and the delivery of mRNA to the egg. Additionally, total RNA extracted from mouse cauda sperm and introduced into parthenotes through microinjection has been shown to regulate ∼10% of genes in the early embryo [45]. However, delineation of the impact of different sperm RNA fractions has not been explored. We next examined whether big RNAs present in sperm can modulate embryonic gene expression, similar to the established post-fertilization functions of sperm small RNAs [15]. To address this, we microinjected parthenotes with either control RNA (H3.3-GFP mRNA alone), total, or big RNA (>200 nucleotides) extracted from mature mouse cauda epididymal sperm (Supplementary Fig. S5A). Parthenotes are chemically activated eggs that undergo development in the absence of a sperm cell, allowing for the unique detection of the function of sperm RNAs. Injected parthenotes were cultured and collected at the four-cell and morula stage for single-embryo RNA-seq, along with biparental (sperm-fertilized) embryos generated via IVF with eggs from the same cohort of superovulated females used for parthenogenesis (Fig. 7A).

This analysis revealed large transcriptomic changes between control parthenotes, and IVF-generated embryos at both developmental stages, as expected. Notably, the introduction of either sperm total or big RNA led to the differential expression of genes in parthenotes at the four-cell and morula stage, compared to control (Fig. 7B and C; Supplementary Fig. S5B and C). The number of shared DEGs between total and big RNA-injected parthenotes compared to controls was 72 and 95 for four-cell and morula stage embryos, accordingly (Supplementary Fig. S5E and F). Importantly, we identified 151 and 193 shared DEGs in parthenotes supplemented with total RNA and IVF embryos when compared to control parthenotes at the four-cell and morula stage, respectively (Supplementary Fig. S5B and C, blue dots), indicating that sperm RNA supplementation can program parthenote gene expression to be more embryo-like. Overall, the correlation of gene expression between sperm total RNA injections and IVF embryos compared to control injected parthenotes was *r* = 0.16 in four-cell and *r* = 0.08 in morula embryos, indicating wide-ranging alterations in gene expression modulated by sperm RNAs that mimic fertilization. Further, when sperm big RNA was injected, the correlation increased to *r* = 0.26 and *r* = 0.09 for four-cell and morula, respectively. Indeed, 139 genes were equally altered in big RNA-injected parthenotes and IVF embryos compared to control parthenotes at the four-cell stage, while at the morula stage we identified 227 shared DEGs, demonstrating that for many genes the introduction of sperm RNA >200 nucleotides resulted in a parthenote’s transcriptional profile more closely resembling that of IVF-derived embryos.

Comparison of DEGs across all datasets revealed 32 genes shared between IVF, total, and big RNA-injected parthenotes compared to control parthenotes at the four-cell stage, while 43 genes were shared at the morula stage (Fig. 7D–F). Consistent with delivery of sperm small RNA (included in total RNA), total RNA-injected parthenotes displayed a reduced expression of a subset of genes previously identified to be regulated by sperm small RNAs during preimplantation embryonic development, which were not regulated when sperm big RNA was supplemented (Supplementary Fig. S5G and H). Importantly, parthenotes injected with big RNA shared more DEGs with IVF embryos than total RNA-injected parthenotes (Supplementary Fig. S5D), indicating these RNAs can indeed modulate postfertilization embryonic gene expression.

## Discussion

Sperm harbor a diverse profile of RNA, including coding and non-coding RNAs. The latter, specifically small non-coding RNAs, have been demonstrated to causally transmit environmentally modulated non-genetically inherited phenotypes to offspring [46, 47]. Conversely, while mRNAs were among the first RNA reported in sperm, they have received comparatively little investigation [1]. This is likely due, in part, to the incongruity between the relatively low abundance of sperm RNA compared to the large amount of RNA stored in the egg, as well as the assumption that mRNAs detected in sperm are merely remnants of late-stage spermatogenesis. However, emerging evidence has begun to identify post-fertilization roles for specific mRNA transcripts [11, 12]. Here, we sought to determine whether, like sperm proteins and small RNAs, mRNAs are dynamically modulated during epididymal transit and to define the role of EVs in this process. Our analysis revealed dynamic changes in mRNAs isolated from sperm, epithelial cells, and EVs along the epididymis, with sperm exhibiting the most pronounced shifts in mRNA profiles from the caput to cauda epididymis. Moreover, we confirm the transfer of mRNA to sperm via epididymal EVs *in vitro* and importantly demonstrate that > 200 nucleotide RNAs delivered by sperm to the zygote are active post-fertilization and are associated with the regulation of embryonic gene expression.

### Epididymal contribution to the mRNA profile of spermatozoa

Our data reveal that sperm epididymal maturation is accompanied by modulation of the sperm mRNA profile. Interestingly, this modulation sees the loss of >1300 mRNAs in sperm from the caput epididymis to the cauda (Fig. 2A). While not yet experimentally validated, the loss of macromolecules from sperm has been attributed to the shedding of the residual cytoplasm (in the form of the cytoplasmic droplet) that occurs early in the epididymis [48]. Conversely, ~1000 mRNAs increase in abundance in cauda epididymal sperm compared to caput epididymal sperm. The predominance of mRNA loss over gain in sperm during epididymal transit mirrors patterns observed in their proteomic cargo [20]. The main form of intercellular communication in the epididymis, responsible for delivering proteins and small RNAs to epididymal sperm, is EVs. Resulting from the transcriptionally and translationally silent state of epididymal sperm, this form of intercellular communication represents a mechanism that shapes the sperm macromolecular cargo, facilitating sperm maturation. Indeed, we demonstrate here that epididymal EVs also transfer mRNAs to sperm (Fig. 3A). Co-incubation of caput epididymal sperm with cauda epididymal EVs increased the abundance of 319 mRNAs, 77 of which are exclusively expressed in cauda epididymal sperm. Moreover, 217 mRNAs were reduced in caput epididymal sperm co-incubated with EVs compared to control caput epididymal sperm. Interestingly, this observation suggests a loss of mRNA during *in vitro* co-incubation, potentially reflecting active molecular exchange. Indeed, EVs have been proposed to facilitate the bidirectional transfer of molecules to refine the sperm macromolecular cargo, however this is yet to be experimentally validated [49]. While these changes do not encompass the breadth of alterations that occur in sperm from caput to cauda epididymis *in vivo*, they demonstrate the ability of EVs to transfer big RNA species (>200 nucleotides), larger than small RNAs, to sperm. The additional time spent in contact with EVs *in vivo* (7–10 days compared to 2 h *in vitro*) together with additional modes of intracellular communication further shapes the sperm mRNA profile. Indeed, nanotubules, the delivery of RNA via RNA-binding proteins, and the shuttling of RNA from the cytoplasmic droplet are all mechanisms of refining the sperm macromolecular cargo that could contribute to the sperm mRNA profile modulation [26, 27, 50]. Overall, examination of the mRNA profiles along the epididymis highlighted three key findings: (i) sperm are a specialized cell with a large number of unique transcripts that are not shared with epididymal epithelial cells or EVs and therefore likely remnants of germline transcription occurring at the culmination of spermatogenesis, (ii) the EV mRNA profile is less complex than their parent cell (epithelial cells), and (iii) RNA within EVs is also present in either sperm or epithelial cells.

In line with previous reports, it is also evident that sperm retain transcripts originating from earlier stages of spermatogenesis. Indeed, several highly abundant mRNAs in cauda epididymal sperm are well-known spermatogenic transcripts that encode proteins required for sperm to function and thus important for male fertility. For example, *Spa17* encodes a protein (SP17) involved in sperm-zona pellucida interaction, while *Smcp* encodes a mitochondria-associated cysteine-rich protein that has been shown to enhance sperm motility [51, 52]. The presence of such transcripts at high abundance in mature sperm suggests that many transcripts are indeed remnants from spermatogenic gene expression during testicular development. However, we found that *Smcp* is significantly increased in abundance in cauda epididymal sperm compared to caput epididymal sperm, an expression pattern also seen in epididymal tissue [28]. Thus, genes typically expressed in the testis also exhibit dynamic levels in epididymal sperm, suggesting that germline mRNAs are regulated within sperm during epididymal transit. Interestingly, while abundant in cauda epididymal EVs, *Smcp* is not detected in either caput or cauda epididymal epithelial cell preparations. Because epididymal epithelial cell preparations involve incubation of single-cell suspensions, these samples are unlikely to retain proton-secreting epithelial cells (clear cells), a population that expresses *Smcp* and produces their own population of epididymal EVs [26]. Overall, our finding suggests that epididymal EVs also contain spermatogenic expressed mRNAs. This supports the notion that EVs may facilitate bidirectional transfer of macromolecules or that the EV composition within the epididymal lumen is more complex than being solely derived from the epididymal epithelium and may, in part, be contributed by sperm. Thus, epididymal luminal EVs may enable RNA exchange extending beyond epithelial-to-sperm transfer.

More simply, our analysis revealed numerous examples of canonical transfer from epididymal EVs to sperm, specifically mRNAs that showed a concomitant increase in both sperm and EVs from the caput to the cauda epididymis (Fig. 2F). Notable examples include *Akr1b7, Azin2*, and *Gpx3*, which encode proteins that are broadly involved in redox regulation and metabolic support for sperm maturation [53, 54]. Moreover, one of the mRNAs most significantly increased in cauda epididymal sperm compared to caput epididymal sperm was *Crisp1*. This mRNA is not expressed in the testis, and its protein product CRISP1 is well-known to be secreted from the epididymis and involved in several key sperm functional maturation events [36, 55, 56]. Here, we show abundant *Crisp1* mRNA levels in sperm, EVs, and epithelial cells from the cauda epididymis but low expression in the caput epididymis. CRISP1 protein is also increased in abundance in cauda epididymal sperm, compared to caput epididymal sperm, with its acquisition important for sperm functionality [20, 56]. This increase in CRISP1 protein levels alongside elevated *Crisp1* mRNA in the sperm cell is surprising given the translationally silent state of spermatozoa. Given the inability of sperm to synthesize new proteins, a suggested role for an epididymal contribution to the sperm mRNA profile could be for delivery to the egg at fertilization for translation in the zygote [57]. However, for many sperm mRNAs, this contrasts with the current known functions of their protein products in events surrounding sperm functional maturation, such as motility and sperm-egg interaction. Therefore, further research to understand the functional significance of this subset of sperm-related mRNAs accumulated during epididymal transit should be explored.

### Sperm deliver mRNAs to the zygote

Owing to the lack of translational machinery within epididymal sperm, we hypothesize that sperm mRNAs function post-fertilization upon delivery to the egg cytoplasm, through either translation of the mRNAs or non-coding mechanisms. We identified 368 mRNAs in the zygote that were also abundant in sperm samples but not detected in the egg. Importantly, a portion of these mRNAs were also enriched in cauda epididymal sperm and delivered to caput epididymal sperm through EV co-incubation (Fig. 5C). Thus, these findings demonstrate that somatically derived mRNAs from the epididymis are delivered to the zygote, positioning them to function post-fertilization and influence early embryonic development. Of the mRNAs exclusive to sperm and zygotes was *Cabyr*, the calcium-binding tyrosine phosphorylation-regulated protein-encoded transcript, which has been previously shown to be transferred by sperm to the egg in humans and bulls [58, 59]. The established role of the *Cabyr* protein product is in modulating intercellular calcium levels during capacitation [60]. Thus, its presence at the mRNA level in sperm and delivery to the zygote remain enigmatic.

While our RNA-seq approach enriches for poly(A) RNAs prior to fragmentation, reducing the likelihood of sequencing mRNA fragments, the detection of fragmented transcripts remains a potential limitation. To assess this, we compared our sperm mRNA dataset to a publicly available catalog of sperm intact RNAs (spiRNAs), defined using long-read sequencing and representing full-length mRNA transcripts in mouse and human sperm [10]. Specifically, 3440 spiRNAs were detected in mouse sperm, including 2343 mRNAs. Of the mRNAs identified in mature cauda epididymal sperm in our data, 803 were also identified spiRNAs, including *Cabyr, Smcp, Crisp1*, and *Spa17*. Moreover, our data reveal reads from mRNAs spanning the gene body, a result indicative of full-length mRNAs, rather than mRNA fragments. SpiRNAs were also detected in populations of human sperm, revealing the clinical relevance of this work. Similarly, we demonstrate the conservation of mouse sperm mRNAs in human sperm, suggesting potential conserved functions postfertilization or during sperm maturation (Fig. 6). Notably, a higher proportion of the mRNAs identified as being transmitted to the zygote had human orthologs compared to the overall mouse transcriptome.

### The functional role of sperm big RNAs

Microinjection of environmentally altered sperm RNAs, either isolated total or fractionated small RNAs from sperm populations, or synthetic miRNA, has been demonstrated to facilitate the non-genetic inheritance of offspring phenotypes [4, 61–64]. Moreover, in line with previous reports, we demonstrate here that parthenogenetically activated eggs are a tractable model for isolating the functions of sperm RNAs in the absence of other sperm-derived factors [45]. Indeed, we determined the ability of sperm RNA to regulate gene expression in the early embryo by microinjecting purified total RNA, as well as size selected big RNA (>200 nucleotides) from mature sperm. In doing so, we confirm the downregulation of a subset of genes known to be regulated by sperm small RNAs upon the introduction of sperm total RNA [15]. However, in line with the selection of RNAs >200 nucleotides, and therefore the removal of small RNAs, supplementation of sperm big RNA did not lead to this downregulation (Supplementary Fig. S5H). Further, we show for the first time that zygotic injection of sperm big RNAs can strikingly modulate preimplantation gene expression. Specifically, supplementation of sperm big RNAs into parthenotes programs the expression of 192 genes in four-cell parthenotes to mimic the IVF embryo (Fig. 7B and D). An interesting observation from our microinjection experiments at the four-cell stage is the correlation between gene expression profiles of big RNA-injected parthenotes and IVF embryos was higher than that observed between total RNA-injected parthenotes and IVF embryos. This again demonstrates that big RNAs in sperm can influence post-fertilization embryonic gene expression at a level equal too, or greater than, sperm small RNAs. The enrichment of RNAs > 200 nucleotides would include populations of mRNAs, circRNAs, and lncRNAs. Both circRNAs and lncRNAs have been shown to be altered in sperm in response to environmental cues. Moreover, the consequences of these altered sperm big RNAs have been linked to phenotypes in offspring, including metabolic and behavioral measures in paternal stress exposure models [13, 65]. The extent of which the observed gene regulation following RNA microinjection is a result of delivery of sperm mRNAs or other sperm non-coding RNAs (>200 nt) requires additional experimentation to delineate.

Collectively, our findings reveal a rich mRNA repertoire in mouse sperm influenced by the soma (epididymal epithelium), the transfer of these RNAs to the zygote at fertilization, and a previously underappreciated role of sperm RNAs, outside of small RNAs, in regulating embryonic gene expression. These findings reveal a wide array of new RNAs capable of functioning in RNA-mediated epigenetic inheritance. While we present evidence of post-fertilization functions for sperm RNAs > 200 nucleotides in the regulation of gene expression in the early embryo, this does not preclude alternative functional roles in the male reproductive tract prior to ejaculation or in priming the female reproductive tract. However, our findings reveal a mechanism for the post-testicular modulation of sperm mRNAs by the soma (epididymis), which can be modulated by the environment or under stress conditions to transmit non-genetically inherited information to offspring. Thus, a comprehensive understanding of the role of sperm RNA in inheritance requires extensive analysis of the diverse RNA classes present in sperm and the breadth of non-genetic information they encode.

## Supplementary Material

gkag330_Supplemental_Files

## Data Availability

All data are available in the main text or the supplementary materials. Raw and processed sequencing data discussed in this publication have been deposited in the European Nucleotide Archive and are accessible via the following accession PRJEB101750. The complete code supporting this application, named ‘Epididymal mRNA Explorer, is available on GitHub (https://github.com/N-A-Trigg/EpiRNA) and Zenodo (https://doi.org/10.5281/zenodo.19224891).
