# Supplementary material for: Epididymal extracellular vesicles harbor and convey mRNA to sperm for transfer to zygotes
Source: Nucleic Acids Res. 2026 Apr 20;54(7):gkag330. doi: 10.1093/nar/gkag330 (PMC13092975; doi:10.1093/nar/gkag330)
Supplement: gkag330_Supplemental_Files [file gkag330_supplemental_files.zip › 070426004528_Trigg_25_mRNA_paper_Supplemental_Materials_v2.pdf]

TITLE: Epididymal extracellular vesicles harbor and convey mRNA to sperm for transfer to zygotes.

AUTHORS: Natalie A. Trigg[1,2,3*], Grace S. Lee[1,2,4] Alexis G. Leach[1,2,5], and Colin C. Conine[1, 2*]

***CORRESPONDENCE:** conine@upenn.edu, natalie.trigg@newcastle.edu.au

This PDF file includes:

Figs. S1 to S5

Other Supplementary Materials for this manuscript include the following:

Data S1 to S18

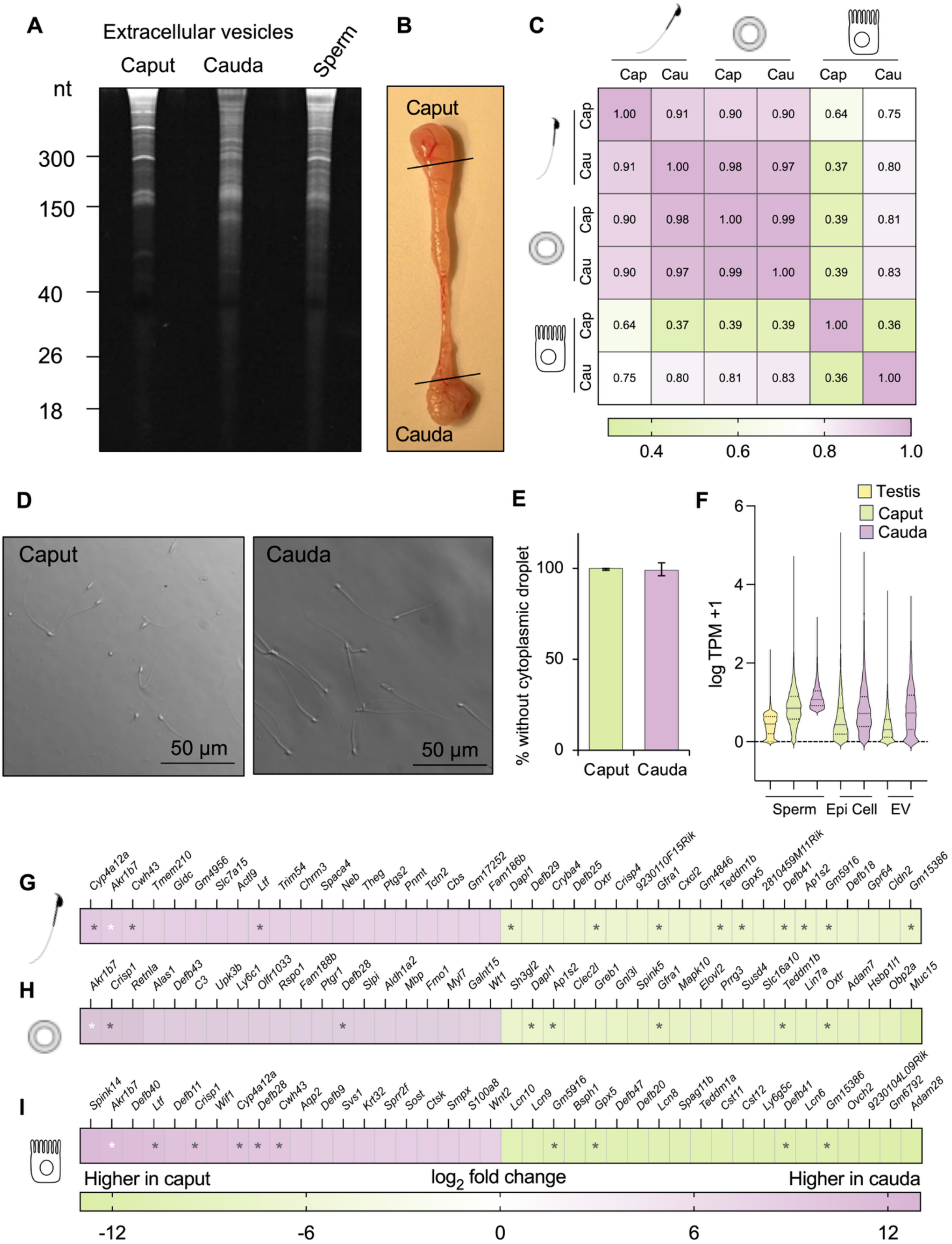

A
Extracellular vesicles
Caput
Cauda
Sperm
nt
300
150
40
26
18
B
Caput
Cauda
C
Cap
Cau
Cap
Cau
Cap
Cau
1.00 0.91 0.90 0.90 0.64 0.75
0.91 1.00 0.98 0.97 0.37 0.80
0.90 0.98 1.00 0.99 0.39 0.81
0.90 0.97 0.99 1.00 0.39 0.83
0.64 0.37 0.39 0.39 1.00 0.36
0.75 0.80 0.81 0.83 0.36 1.00
0.4
0.6
0.8
1.0
D
Caput
Cauda
50 µm
50 µm
E
% without cytoplasmic droplet
100
50
0
Caput
Cauda
F
Testis
Caput
Cauda
log TPM +1
6
4
2
0
Sperm
Epi Cell
EV
G
Cyp4a12a Akr1b7 Cwh43 Tmem210 Gldc Gm4956 Slc7a15 Actl9 Ltf Trim54 Chrm3 Spaca4 Neb Theg Ptgs2 Pnmt Tctn2 Cbs Gm17252 Fam186b Dapl1 Defb29 Cryba4 Defb25 Oxtr Crisp4 9230110F15Rik Gfra1 Cxcl2 Gm4846 Teddm1b Gpx5 2810459M11Rik Defb41 Ap1s2 Gm5916 Defb18 Gpr64 Cldn2 Gm15386
H
Akr1b7 Crisp1 Retnla Alas1 Defb43 C3 Upk3b Ly6c1 Olfr1033 Rspo1 Fam188b Ptgr1 Defb28 Slpi Aldh1a2 Mbp Fmo1 Myl7 Galnt15 Wt1 Sh3gl2 Dapl1 Ap1s2 Clec2l Greb1 Gnl3l Spink5 Gfra1 Mapk10 Elovl2 Prrg3 Susd4 Slc16a10 Teddm1b Lin7a Oxtr Adam7 Hsbp1l1 Obp2a Muc15
I
Spink14 Akr1b7 Defb40 Ltf Defb11 Crisp1 Wif1 Cyp4a12a Defb28 Cwh43 Aqp2 Defb9 Svs1 Krt32 Sprr2f Sost Ctsk Smpx S100a8 Wnt2 Lcn10 Lcn9 Gm5916 Bsph1 Gpx5 Defb47 Defb20 Lcn8 Spag11b Teddm1a Cst11 Cst12 Ly6g5c Defb41 Lcn6 Gm15386 Ovch2 9230104L09Rik Gm6792 Adam28
Higher in caput
log2 fold change
Higher in cauda
-12
-6
0
6
12

Figure S1: Epididymal sperm, extracellular vesicles and epithelial cell RNA-seq.

A) Total RNA isolated extracellular vesicles isolated from the caput, and cauda epididymis and from populations of cauda sperm and separated using denaturing PAGE. Gel is stained with SYBR gold to stain nucleic acids. B) Image of a mouse epididymis highlighting the dissected epididymal segments representing the caput and cauda throughout this study. C) Pearsons correlation heatmap of sperm, epithelial cells, and extracellular vesicles from the caput and cauda epididymis. D) Phase microscopy images of purified caput and cauda sperm preparations following somatic cell lysis illustrating negligible somatic cell contamination. E) Graphical representation of the percentage of sperm devoid of cytoplasmic droplets in caput and cauda sperm preparations. F) Violin plot illustrating the abundance (log TPM + 1) of the 2,500 mRNAs detected in cauda epididymal sperm but not in testicular sperm across sperm, epithelial cells, and EVs G-I) Heat maps depicting the $\log_2$ fold change of the top 20 up- and 20 down- regulated mRNAs in cauda compared to caput in G) sperm, H) extracellular vesicles and I) epithelial cells. Asterisks indicate mRNAs that are also differentially expressed (DE) between caput and cauda in other sample types. Grey asterisks = DE in one other sample type, white asterisks = DE across all three sample types.

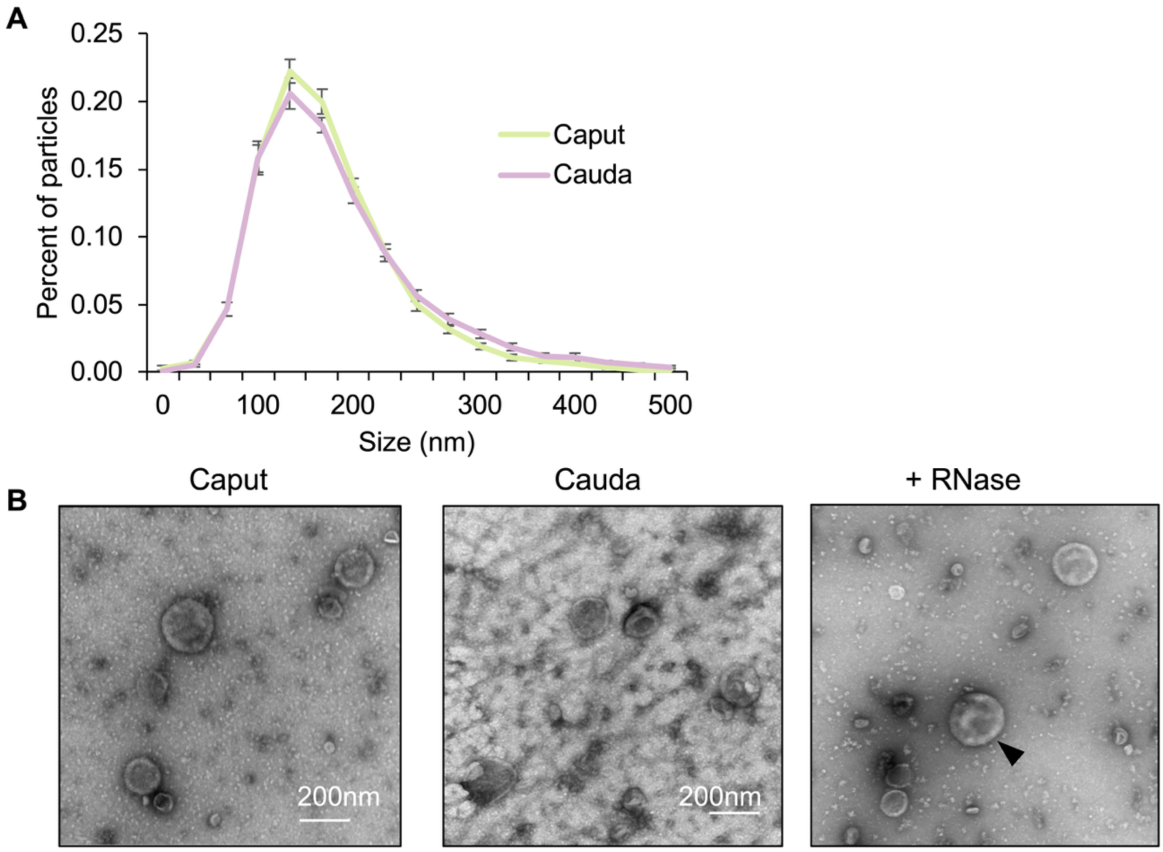

Figure S2: Characterization of epididymal extracellular vesicles.

A) Vesicle size and distribution of caput and cauda extracellular vesicles (EVs). Data was obtained from ZetaView nanoparticle tracking software and is represented as mean with standard error of the mean. B) Negative stain electron microscopy images of EVs isolated from the caput and cauda epididymis and EVs post RNase treatment to visualize vesicle integrity following treatment. Scale bar = 200 nm.

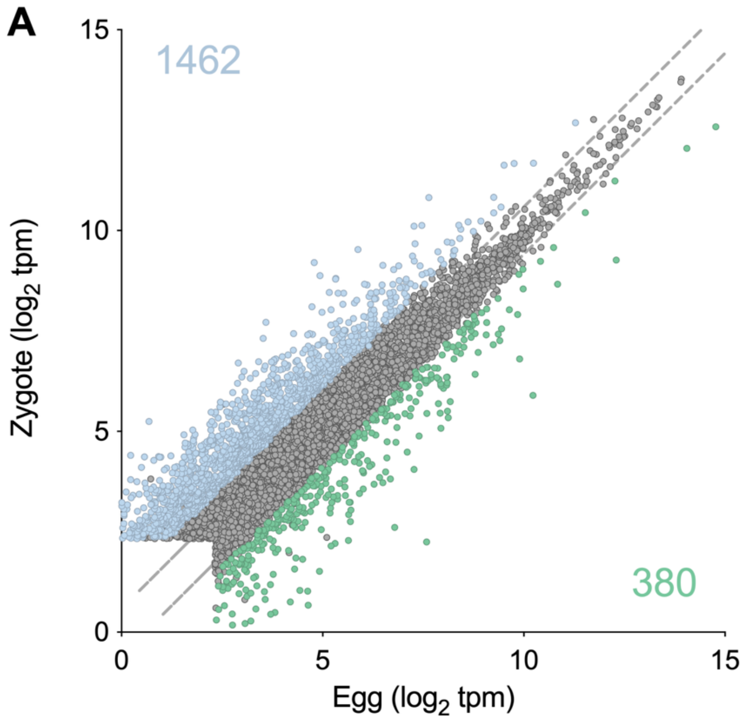

Figure S3: Transcriptomic alterations in mouse zygotes. A) Scatter plot illustrating average $\log_2$ gene expression (transcript per million; TPM) in mouse eggs (x-axis) and fertilized eggs (zygotes; y-axis). Colored dots indicate significantly altered genes, blue indicates genes higher in zygotes while green depicts higher expression in eggs. Threshold for differential expression was fold-change ± 2 and adjusted *P*-value of ≤ 0.01.

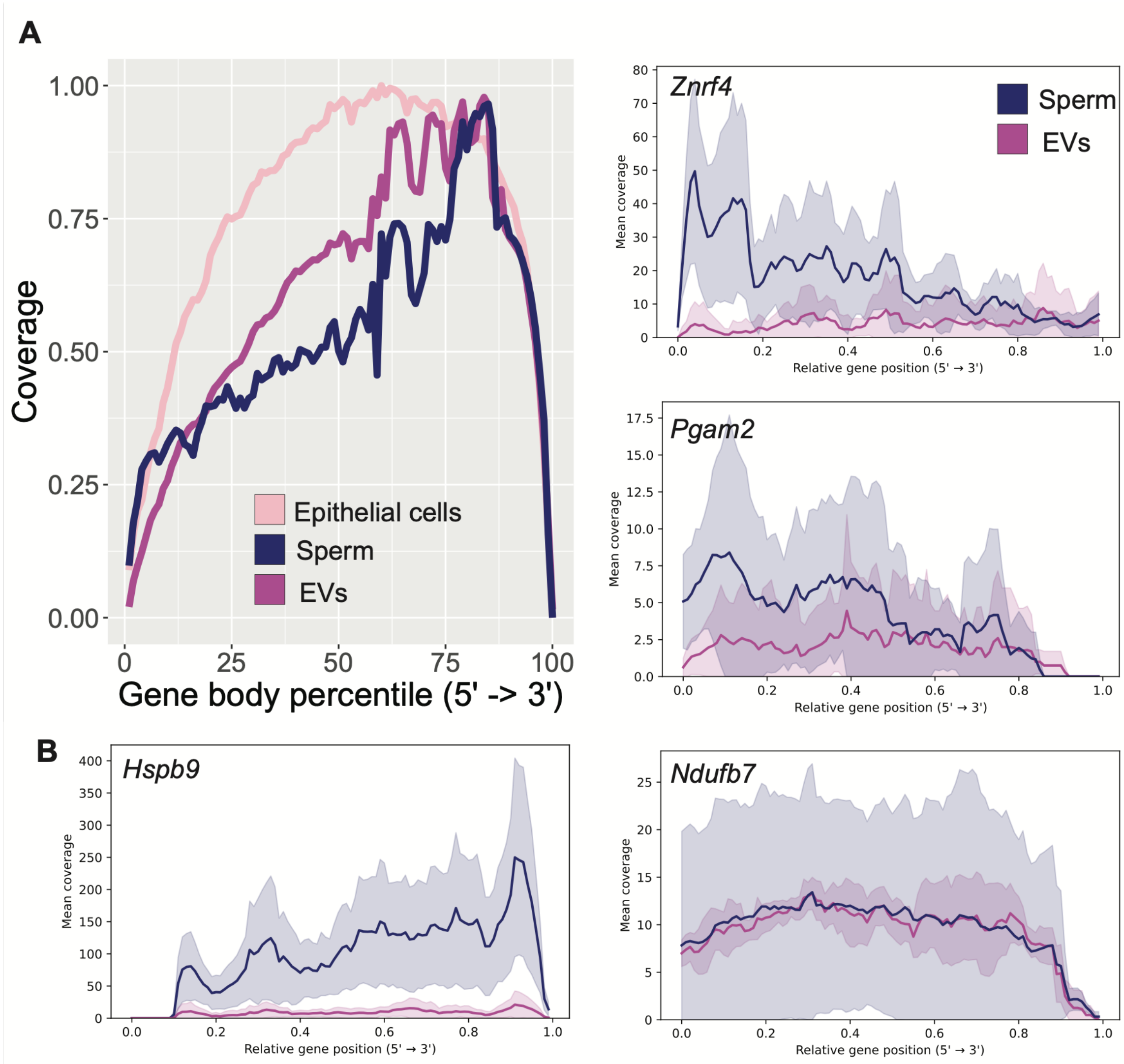

Figure S4: Gene body coverage of sperm mRNAs. A) Gene body coverage profiles of all mRNAs in epithelial cells, sperm, and EVs isolated from the cauda epididymis. Each line represents the meta-gene average coverage across all mRNAs (excluding introns) in the sample. B) Gene coverage plots for individual sperm mRNAs (exons only) showing the 5'-3' distribution of reads in sperm and EV samples from the cauda epididymis. Lines illustrates mean coverage and shading indicates standard deviation.

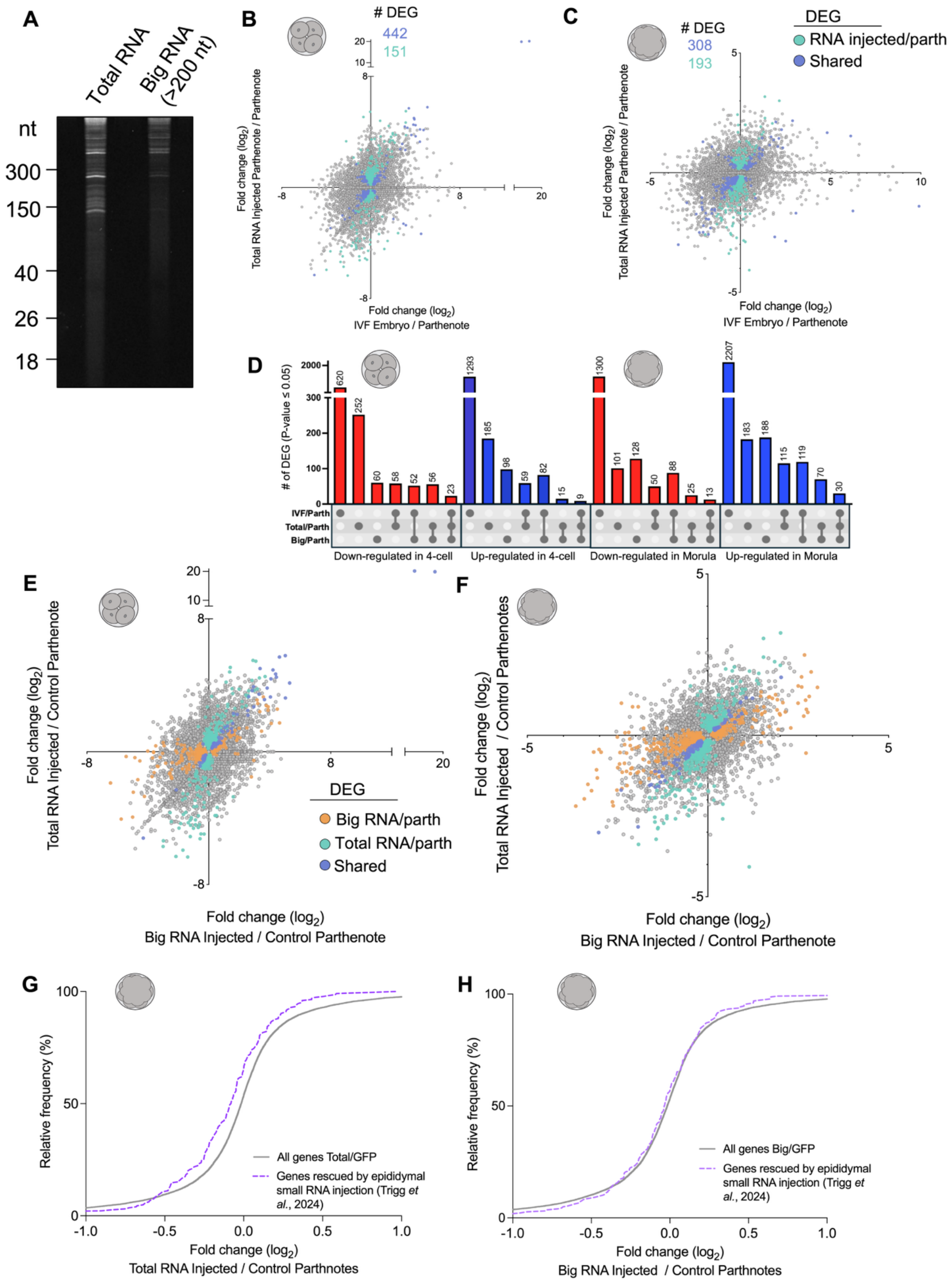
A
Total RNA
Big RNA (>200 nt)
nt
300
150
40
26
18
B
DEG
442
151
Fold change (log2) Total RNA Injected Parthenote / Parthenote
Fold change (log2) IVF Embryo / Parthenote
C
DEG
308
193
DEG
RNA injected/parth
Shared
Fold change (log2) Total RNA Injected Parthenote / Parthenote
Fold change (log2) IVF Embryo / Parthenote
D
# of DEG (P-value ≤ 0.05)
IVF/Parth
Total/Parth
Big/Parth
Down-regulated in 4-cell
Up-regulated in 4-cell
Down-regulated in Morula
Up-regulated in Morula
E
Fold change (log2) Total RNA Injected / Control Parthenote
Fold change (log2) Big RNA Injected / Control Parthenote
DEG
Big RNA/parth
Total RNA/parth
Shared
F
Fold change (log2) Total RNA Injected / Control Parthenotes
Fold change (log2) Big RNA Injected / Control Parthenote
G
Relative frequency (%)
All genes Total/GFP
Genes rescued by epididymal small RNA injection (Trigg et al., 2024)
Fold change (log2) Total RNA Injected / Control Parthnotes
H
Relative frequency (%)
All genes Big/GFP
Genes rescued by epididymal small RNA injection (Trigg et al., 2024)
Fold change (log2) Big RNA Injected / Control Parthnotes

Figure S5: Sperm RNAs influence gene expression in parthenotes. A) Total RNA and size selected big RNA (>200 nucleotides) from cauda epididymal sperm preparations were separated using denaturing PAGE and stained with SYBR gold to stain nucleic acids. B-C) Correlation plots comparing the $\log_2$ fold change of genes between IVF produced embryos and parthenotes (x-axis) to total RNA injected parthenotes and control parthenotes. Data for (B) 4-cell and (C) morula. Colored dots indicate differentially expressed genes (DEGs) for total RNA injected parthenotes compared to control parthenotes (blue), and those that were similarly altered in IVF embryos compared to control parthenotes (purple). D) Upset plot depicting DEG sets in 4-cell and morula embryos for all comparisons. Colored bars indicate the fold change direction, where red indicates increased abundance compared to control parthenotes and blue indicates decreased abundance. E-F) Correlation plots comparing the $\log_2$ fold change of genes between big RNA and control injected parthenotes (x-axis) to total RNA and control injected parthenotes. Data for (E) 4-cell and (F) morula. Colored dots indicate differentially expressed genes (DEGs) for big RNA injected versus control parthenotes (orange), total RNA injected compared to control parthenotes (blue), and those that were similarly altered in both groups (purple). G-H) Cumulative distribution plot for $\log_2$ fold change in morula embryos for G) total and H) big RNA injected parthenotes compared to control parthenotes. Lines depicting all genes (gray) for total RNA injected versus control injected and big RNA injected versus control injected. Gene subsets are examined in all CDF plots with purple dotted lines demonstrating genes previously identified to be rescued in mutant embryos by injection of epididymal small RNAs, which would be included in the total RNA injection.

Supplemental Table S1: Transcript per million (TPM) of detected RNA transcripts across biological replicates of mouse sperm isolated from the caput or cauda epididymis. Gene lists have been filtered to obtain a list of detected transcripts. This filtering involved removal of any transcripts with less than 5 TPM average and removal of transcripts with less than half of replicates exhibiting TPM ≤ 2. This table consists of genes that satisfy these criteria for at least one of the groups (caput or cauda).

Supplemental Table S2: Comparative analysis of mRNAs detected in mouse sperm isolated from the caput and cauda epididymis of untreated mice was completed using DESeq2 package in R. Genes were defined as differentially abundant when they met the criteria of a fold change ≥ 2 or ≤ -2, and an adjusted P-value < 0.05.

Supplemental Table S3: Transcript per million (TPM) of detected RNA transcripts across biological replicates of mouse sperm isolated from the caput epididymis and mock incubated or co-incubated with extracellular vesicles isolated from the cauda epididymis (cap sp plus). Gene lists have been filtered to obtain a list of detected transcripts. This filtering involved removal of any transcripts with less than 5 TPM average and removal of transcripts with less than half of replicates exhibiting TPM ≤ 2. This table consists of genes that satisfy these criteria for at least one of the groups (caput or cauda).

Supplemental Table S4: Comparative analysis of mRNAs detected in mouse caput sperm mock incubated or co-incubated with extracellular vesicles isolated from the cauda epididymis was completed using DESeq2 package in R. Genes were defined as differentially abundant when they met the criteria of a fold change ≥ 1.5 or ≤ -1.5, and a P-value < 0.05.

Supplemental Table S5: Transcript per million (TPM) of detected RNA transcripts across biological replicates of mouse epithelial cells isolated from the caput or cauda epididymis. Gene lists have been filtered to obtain a list of detected transcripts. This filtering involved removal of any transcripts with less than 5 TPM average and removal of transcripts with less than half of replicates exhibiting TPM ≤ 2. This table consists of genes that satisfy these criteria for at least one of the groups (caput or cauda).

Supplemental Table S6: Comparative analysis of mRNAs detected in epithelial cells isolated from the caput and cauda epididymis of untreated mice was completed using DESeq2 package in R. Genes were defined as differentially abundant when they met the criteria of a fold change ≥ 2 or ≤ -2, and an adjusted P-value < 0.05.

Supplemental Table S7: Transcript per million (TPM) of detected RNA transcripts across biological replicates of extracellular vesicles (EVs) isolated from the caput or cauda epididymis. Gene lists have been filtered to obtain a list of detected transcripts. This filtering involved removal of any transcripts with less than 5 TPM average and removal of transcripts with less than half of replicates exhibiting TPM ≤ 2. This table consists of genes that satisfy these criteria for at least one of the groups (caput or cauda).

Supplemental Table S8: Comparative analysis of mRNAs detected in extracellular vesicles (EVs) isolated from the caput and cauda epididymis of untreated mice was completed using DESeq2 package in R. Genes were defined as differentially abundant when they met the criteria of a fold change ≥ 2 or ≤ -2, and an adjusted P-value < 0.05.

Supplemental Table S9: Transcript per million (TPM) of detected RNA transcripts across biological replicates of extracellular vesicles (EVs) isolated from the caput epididymis and either mock treated or treated with RNases. Gene lists have been filtered to obtain a list of detected transcripts. This filtering involved removal of any transcripts with less than 5 TPM average and those detected in only a single replicate (of three). This table consists of genes that satisfy these criteria for at least one of the groups and detected in untreated EV samples.

Supplemental Table S10: Transcript per million (TPM) of detected RNA transcripts across biological replicates of extracellular vesicles (EVs) isolated from the cauda epididymis and either mock treated or treated with RNases. This filtering involved removal of any transcripts with less than 5 TPM average and those detected in only a single replicate (of three). This table consists of genes that satisfy these criteria for at least one of the groups and detected in untreated EV samples.

Supplemental Table S11: Transcript per million (TPM) of detected RNA transcripts across individual eggs collected from control female mice and zygotes generated with gametes from control male and female mice. Gene lists have been filtered to remove any transcripts with less than 5 TPM average in at least one group (egg or zygote).

Supplemental Table S12: Comparative analysis of mouse MII eggs and zygote gene expression profiles derived from untreated mice. Individual eggs and zygotes were sequenced and comparative analysis using DESeq2 package in R was completed. Genes were defined as differentially expressed when they met the criteria of a fold change ≥ 2 or ≤ -2, and an adjusted P-value < 0.05.

Supplemental Table S13: Average transcript per million (TPM) of 803 mRNAs detected in our cauda sperm dataset and identified in previously published dataset of sperm intact RNAs (Sun et al., 2021).

Supplemental Table S14: Average transcript per million (TPM) of mRNAs detected in mouse cauda sperm and with identified human orthologs.

Supplemental Table S15: Transcript per million (TPM) of detected RNA transcripts across individual 4-cell embryos cultured from naive IVF zygotes (IVF) or parthenotes microinjected with control GFP RNA (GFP), total RNA isolated from mouse sperm (total), and big RNA (> 200 nucleotides) size selected from RNA isolated from mouse sperm. Gene lists have been filtered to remove any transcripts with less than 5 TPM average in at least one group.

Supplemental Table S16: Transcript per million (TPM) of detected RNA transcripts across individual morula embryos cultured from naive IVF zygotes (IVF) or parthenotes microinjected with control GFP RNA (GFP), total RNA isolated from mouse sperm (total), and big RNA (> 200 nucleotides) size selected from RNA isolated from mouse sperm. Gene lists have been filtered to remove any transcripts with less than 5 TPM average in at least one group.

Supplemental Table S17: Differentially expressed genes (DEG) in 4-cell and morula embryos across different comparisons. Comparative analysis was completed using DESeq2 package in R. Genes were defined as differentially expressed when they met the criteria of a fold change ≥ 2 or ≤ -2, and an adjusted P-value < 0.05. Groups include IVF embryos, parthenotes injected with total RNA, or big RNA (>200 nucleotide) from mouse sperm compared to control group (parthenotes injected with GRP RNA). Final column in each table indicates whether the DEG is shared across both comparisons or unique.

Supplemental Table S18: Transcript per million (TPM) of detected RNA transcripts across biological replicates of testicular sperm. Gene lists have been filtered to obtain a list of detected transcripts. This filtering involved removal of any transcripts with less than 5 TPM average and those detected in only a single replicate (of three). This table consists of genes that satisfy these criteria.
